# Self‐Healing Ionogel Dual‐Network Enables Synergistic Defect Passivation and Strain Relief for High‐Efficiency Robust Perovskite Photovoltaics

**DOI:** 10.1002/advs.77804

**Published:** 2026-09-27

**Authors:** Wen Wu, Gang Wang, Chen Liu, Xiao‐Li Du, Sheng Yang, Shao‐Long Wang, Jia‐Yi Tian, Ling‐Feng Chao, Xiang‐Feng Deng, Yi Jiang, Yong‐Hua Chen, Gui‐Chuan Xing, Wen‐Yong Lai

**Affiliations:** ^1^ State Key Laboratory of Flexible Electronics (LoFE) Institute of Advanced Materials (IAM) School of Chemistry and Life Sciences Nanjing University of Posts & Telecommunications Nanjing China; ^2^ Joint Key Laboratory of the Ministry of Education Institute of Applied Physics and Materials Engineering University of Macau Taipa Macao SAR China; ^3^ State Key Laboratory of Flexible Electronics (LoFE) and Institute of Advanced Materials (IAM) School of Flexible Electronics (Future Technologies) Nanjing Tech University Nanjing China

**Keywords:** defect and residual strain, efficiency and stability, flexible photovoltaics, ionogel, perovskite solar cells

## Abstract

The simultaneous realization of high efficiency and long‐term stability remains the central challenge for the commercialization of perovskite photovoltaics. The solution processing of perovskite films, while offering significant advantages, inevitably introduces deep‐level defects and residual strain, fundamentally limiting both the achievable open‐circuit voltage and the device's operational durability. Here, we introduce a molecular design concept that concurrently addresses these pervasive issues by engineering a cross‐linked multifunctional ionogel, [HA‐AA][EMIES], which is incorporated into the precursor solution. This approach leverages the ionogel's extensive dynamic bond networks and its intrinsic matrix toughening mechanism, achieved through molecular‐scale bond energy dissipation, to orchestrate the crystallization process and manage mechanical stress. Consequently, we achieve a near two‐fold enhancement in carrier diffusion length, surpassing 8 µm within the polycrystalline film. The resulting [HA‐AA][EMIES]‐based devices demonstrate superior efficiencies of 26.45% for rigid substrates and 25.14% for flexible devices, while exhibiting exceptional resilience against both ambient air exposure and mechanical bending. This work establishes a direct route to eliminate deleterious defects and residual strain in perovskites, offering a promising pathway to accelerate the development and commercial deployment of large‐area, stable perovskite photovoltaics.

## Graphical abstract

PSCs are afflicted by inherent defects and lattice strain that limit PCE and stability. A cross‐linked multifunctional ionogel [HA‐AA][EMIES] is developed to modulate crystallization, relieve residual strain, and passivate defects, extending carrier diffusion length to over 8 µm and delivering PCEs of 26.45% for rigid devices and 25.14% for flexible devices with operational and ambient stability.

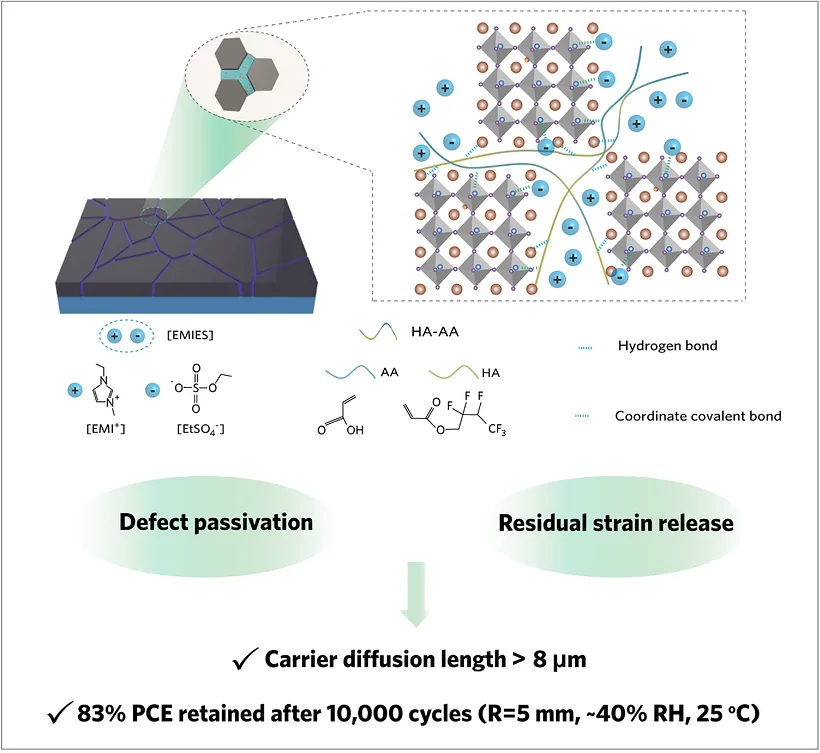

## 1 Introduction

Metal‐halide perovskite solar cells (PSCs) have emerged as a transformative photovoltaic technology, demonstrating remarkable potential for terawatt‐scale renewable energy generation [1, 2]. With power conversion efficiencies (PCEs) for single‐junction devices now surpassing 27% [3], they are rapidly approaching the theoretical Shockley–Queisser limit. The unparalleled advantage of PSCs lies in their compatibility with low‐temperature solution processing, offering a compelling pathway toward cost‐effective commercialization [4]. However, the inherently rapid reaction kinetics and ionic diffusion during solution fabrication inevitably introduce abundant defects within the perovskite film [5, 6]. These defects act as non‐radiative recombination centers, trapping charge carriers, and simultaneously induce localized lattice distortions. This distortion manifests as significant internal residual strain within the film. This intrinsic strain is further compounded by external thermal expansion mismatch between perovskite and substrate during annealing [7, 8]. In turn, this accumulated residual strain facilitates the formation of additional defects within the perovskite. The detrimental combination of non‐radiative recombination and residual strain enhances carrier scattering and suppresses long‐range carrier diffusion [9], critically limiting both the achievable PCE and the long‐term operational stability of PSCs [10, 11, 12]. Therefore, a strategic approach that synergistically orchestrates the crystallization process to enhance film uniformity and releases residual strain to prolong carrier diffusion lengths is imperative to unlock the full commercial potential of PSCs.

Modulating the precursor chemistry is a powerful strategy to govern the film‐forming process, where the pathway from the solution state to the final polycrystalline film dictates crystal nucleation and growth [5, 7, 13]. Conventional additives, such as organic ammonium salts (e.g., methylammonium chloride, MACl) or mixed A‐site cations (e.g., caesium, rubidium), are widely employed to regulate crystallization [14, 15, 16, 17, 18]. However, these approaches often introduce unintended lattice distortion and phase segregation. The inherent volatility and instability of organic ammonium cations, coupled with the size mismatch of A‐site cations, inevitably generate or exacerbate intrinsic strain within the perovskite lattice [19, 20, 21]. Ionic liquids have emerged as promising candidates due to their tunable dynamic bonding, which can modulate nucleation kinetics and promote homogeneous film formation [22, 23]. Their large steric hindrance and high chemical inertness can stabilize the perovskite lattice and passivate defects in situ [24]. Despite these advantages, ionic liquids typically lack a continuous, 3D network structure. This prevents them from effectively dissipating the mechanical strain accumulated at grain boundaries, a critical deficiency for applications in flexible PSCs (*f*‐PSCs) where mechanical durability is paramount [10, 25, 26]. Consequently, a molecular design concept that synergistically couples the dynamic coordination ability of ionic liquids for defect passivation with an elastomeric network for effective strain compensation remains elusive.

Ionogels, which consist of an ionic liquid immobilized within a polymer matrix, have found widespread application in flexible electronics due to their exceptional stretchability, adhesiveness, and self‐healing properties [27, 28]. These characteristics originate from their abundant dynamic bond networks and the matrix toughening mechanism achieved through bond energy dissipation at the molecular scale [29, 30]. These properties position ionogels as ideal candidates for modulating perovskite film morphology and achieving the critical goal of strain compensation. However, a rational design strategy for ionogels tailored for perovskite film engineering to enhance long‐range carrier diffusion has not yet been explored [31].

**FIGURE 1:**
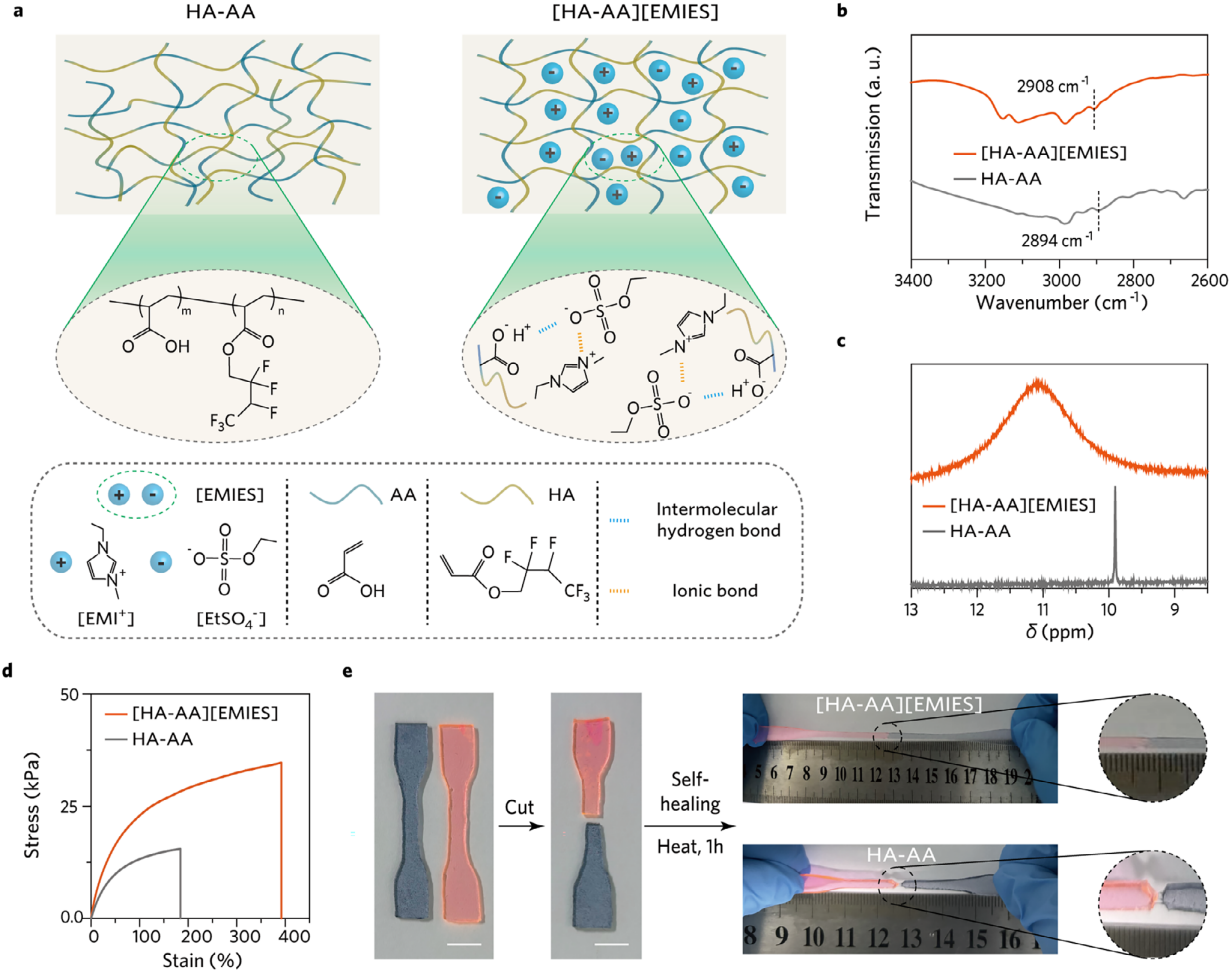
The chemical structure and mechanical properties of HA‐AA and [HA‐AA][EMIES]. (a) Molecular chain states of HA‐AA and [HA‐AA][EMIES]. The segments of HA‐AA tend to arrange randomly, forming a structure analogous to a 3D coil. In [HA‐AA][EMIES], HA‐AA chains are interpenetrated with ionic liquids. In the bottom panel, the molecular structures of [EMI]^+^, [EtSO_4_]^−^, [EMIES], AA and HA groups are presented. The proposed hydrogen and ionic bonds are also depicted. (b, c) The FTIR (b) and ^1^H NMR spectra (c) of HA‐AA and [HA‐AA][EMIES]. (d) Tensile strain curves of HA‐AA and [HA‐AA][EMIES] with a stretched deformation rate of 5 mm min^−1^. (e) The demonstration of self‐healing behaviors of HA‐AA and [HA‐AA][EMIES]. The circular insets show the magnified views of the healed interfaces after stretching. The scale bar in the dog‐bone‐shaped samples (dyed blue and red) is 1 cm.

Herein, we report the design of a novel ionogel, specifically engineered with a fluorine‐rich polar segment to maximize hydrogen‐bond density, which substantially delays crystallization and facilitates the release of residual strain in formamidinium lead halides (FAPbI_3_) films. By incorporating a copolymer, hyaluronic acid‐acrylic acid (HA‐AA), with an ionic liquid, 1‐ethyl‐3‐methylimidazolium ethyl sulfate ([EMIES]), we constructed a multifunctional ionogel, denoted as [HA‐AA][EMIES] (Figure 1a). In this system, intermolecular hydrogen bonds form between the carboxylic acid protons of HA‐AA and the ethyl sulfate anion ([EtSO_4_]^−^), concurrent with the intrinsic ionic interactions within [EMIES]. This multiple hydrogen bonding and strong coordination establish a dual cross‐linked network within the ionogel, providing a powerful toolkit for regulating perovskite crystallization. This ionogel significantly increases the nucleation energy barrier, promoting homogeneous nucleation. Crucially, the ionogel embedded at the perovskite grain boundaries effectively dissipates residual strain, imparting enhanced flexibility and self‐healing capability to the film. Benefiting from the concurrent resolution of defect passivation and residual strain release, the charge transport characteristics are dramatically enhanced. We achieve an exceptionally long carrier diffusion length of up to 8.07 µm. This translates to champion PCEs of 26.45% for rigid PSCs and 25.14% for *f*‐PSCs, alongside excellent operational stability.

## 2 Results and Discussion

### 2.1 Structural and Mechanical Characterization of the Multifunctional Ionogel

The development of an effective additive for perovskite film engineering requires a molecular architecture that can simultaneously modulate crystallization and manage mechanical stress. To this end, we designed a dual cross‐linked ionogel, [HA‐AA][EMIES], synthesized via a straightforward photo‐initiated free radical polymerization (see Methods). The copolymer matrix, poly(2,2,3,4,4,4‐hexafluorobutyl acrylate‐*co*‐acrylic acid) (HA‐AA), was first synthesized with a weight‐average molecular weight of 1.75 × 10^5^ (Figure S1). Critically, the carboxylic acid moieties in acrylic acid (AA) segment are ionizable, conferring excellent compatibility with the ionic liquid, [EMIES] [27, 32, 33]. This compatibility is essential for achieving a homogeneous and stable ionogel network. The [EMIES] component provides the 1‐ethyl‐3‐methylimidazolium ([EMI]^+^) cation and the [EtSO_4_]^−^ anion. By incorporating [EMIES] during the pomerization, we successfully formed a material where the polymer chains are interpenetrated with the ionic liquid, giving rise to our target ionogel, [HA‐AA][EMIES]. Within this network, we hypothesized that intermolecular hydrogen bonds would form between the polymer and the ionic liquid, coexisting with the intrinsic ionic interactions of the ionic liquid itself, thereby creating a dual cross‐linked architecture (Figure 1a). Thermogravimetric analysis (TGA) confirmed the enhanced thermal stability of this network, with [HA‐AA][EMIES] exhibiting a decomposition temperature of 304°C, significantly higher than that of HA‐AA (Figure S2).

To elucidate the specific intermolecular interactions within the ionogel, we performed Fourier transform infrared (FTIR) spectroscopy. As shown in Figure 1b, the stretching vibration peak of the carboxylic acid ─OH group in HA‐AA is observed at 2894 cm^−1^. Upon incorporation of [EMIES] to form the ionogel, this peak shifts to 2908 cm^−1^. This shift is indicative of the formation of stronger hydrogen bonds, where the ionizable hydrogen atoms of AA carboxyl groups act as donors, and the strongly electronegative oxygen atoms of [EtSO_4_]^−^ anion act as acceptors [34]. This interaction was corroborated by ^1^H nuclear magnetic resonance (NMR) spectroscopy, which showed a clear downfield shift in the proton resonance of AA carboxylic acid group upon addition of [EMIES] (Figure 1c). It is worth noting that while hydrogen bonding between the fluorine atoms in HA segment and the carboxylic acid is theoretically possible, the absence of a readily ionizable proton in the solvent‐free HA‐AA makes this interaction negligible. In [HA‐AA][EMIES], its polymer network is saturated with [EMIES], further disfavoring any direct F‐H interactions [35]. Therefore, the primary and most significant interactions are the hydrogen bonds between HA‐AA and [EMIES] and the pre‐existing ionic bonds within [EMIES] itself. This combination establishes the desired dual cross‐linked network, which is anticipated to impart superior mechanical properties.

The mechanical characteristics of [HA‐AA][EMIES] are directly relevant to its ability to manage residual strain in perovskite film. Tensile tests revealed a profound enhancement in mechanical properties upon ionogel formation. HA‐AA exhibited a fracture strain of 180% (Figure 1d). In contrast, [HA‐AA][EMIES] demonstrated exceptional flexibility, with a fracture strain exceeding 390%. This remarkable improvement is attributed to the energy dissipation mechanism provided by its dual network. The abundant hydrogen and ionic bonds act as the reversible and sacrificial bonds that can break and re‐form under stress, effectively absorbing mechanical energy [36, 37].

Beyond its enhanced flexibility, [HA‐AA][EMIES] displayed a notable self‐healing capability, a hallmark of materials with dynamic bond networks. When [HA‐AA][EMIES] was cut in half and the two pieces were brought into contact at 60°C for 1 h, it could healed completely (Figure 1e). In a subsequent tensile test, the healed [HA‐AA][EMIES] exhibited a fracture strain of 225% at 60°C. Notably, [HA‐AA][EMIES] also demonstrated self‐healing capability at room temperature. After 1 h of contact, the rejoined interface remained intact during stretching, yielding a fracture strain of 170% (Figures S3 and S4). Its superior self‐healing ability is direct evidence of the dynamic and reversible nature of the dual cross‐linked network within. This unique combination of flexibility, energy dissipation, and self‐healing positions the [HA‐AA][EMIES] ionogel as an ideal candidate for managing the internal and external stresses that develop during perovskite film formation and operation, particularly in the context of flexible devices. Consequently, we sought to investigate how this multifunctional material could influence the crystallization kinetics and residual strain within the perovskite film itself.

### 2.2 Orchestrating Perovskite Crystallization Through Ionogel‐Mediated Precursor Chemistry

The crystallization pathway of a perovskite film, from the precursor solution to the final polycrystalline solid, is critically dependent on the chemical interactions present during the initial stages of spin‐coating and annealing. To understand how our designed ionogel ([HA‐AA][EMIES]) influences this process, we incorporated HA‐AA and [HA‐AA][EMIES] into the perovskite precursor solution at 2.0 wt.% relative to lead iodide (PbI_2_) component. The resulting perovskite films are hereafter denoted as PVK‐HA‐AA and PVK‐[HA‐AA][EMIES] correspondingly, while the perovskite film without additive used as reference is named as PVK. Scanning electron microscopy (SEM) confirmed that [HA‐AA][EMIES] is preferentially located at the grain boundaries (Figure S5), suggesting its active role in mediating grain growth and interconnection. To further elucidate the spatial distribution of [HA‐AA][EMIES], high‐resolution transmission electron microscopy (HR‐TEM) was performed on PVK and PVK‐[HA‐AA][EMIES]. No amorphous phase is observed at the edge of PVK, whereas PVK‐[HA‐AA][EMIES] exhibits a continuous amorphous phase at the edges of crystalline domains (Figure S6a,b). At the internal grain boundaries (Figure S6c,d), PVK retains clear lattice fringes with spacing of 1.54 and 1.57 Å, while a distinct amorphous phase appears between adjacent crystalline grains in PVK‐[HA‐AA][EMIES]. These observations strongly support the preferential accumulation of [HA‐AA][EMIES] at the grain boundaries.

**FIGURE 2:**
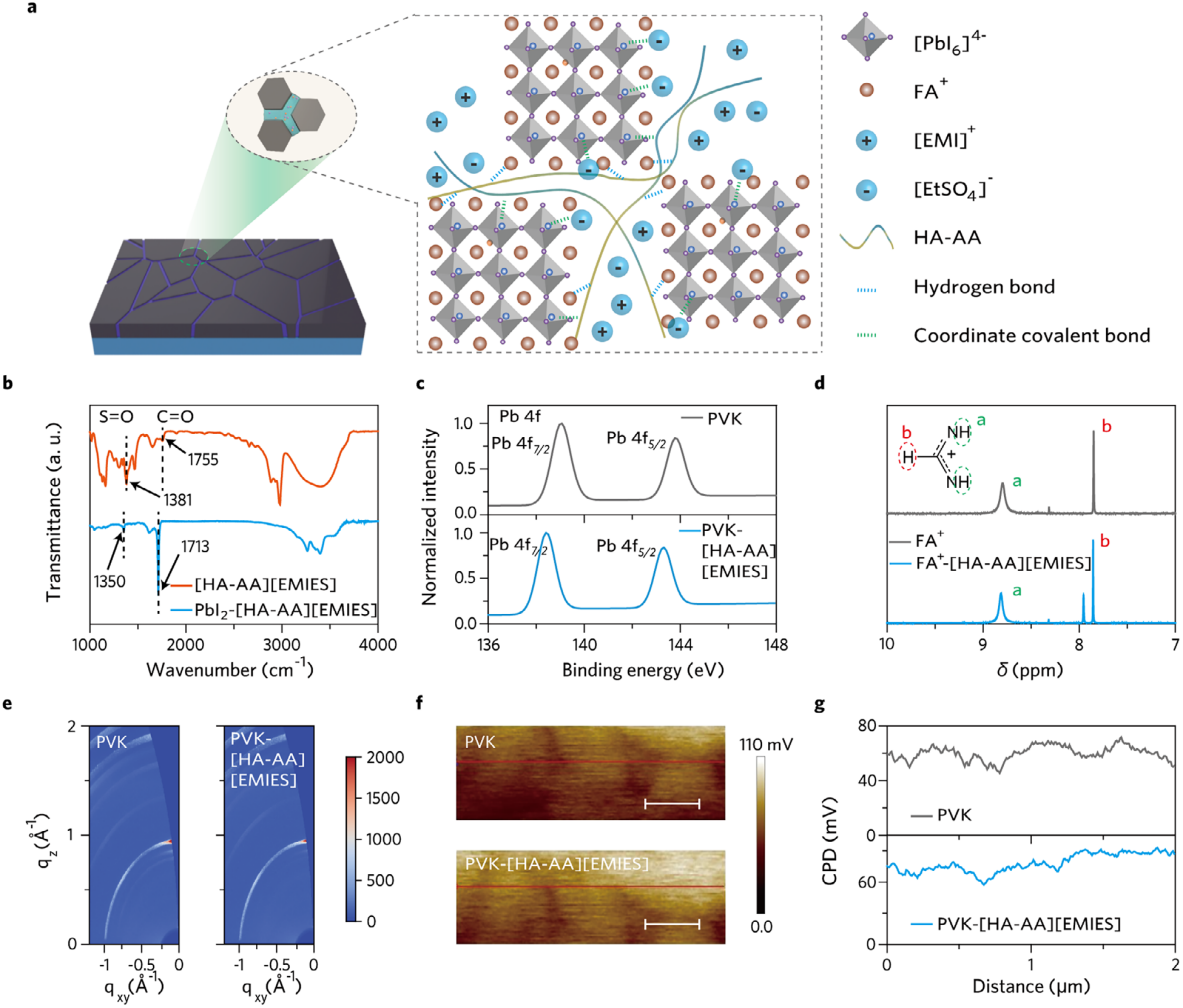
Effects of [HA‐AA][EMIES] on the crystallization of perovskite films. (a) Schematic diagram of the interactions between [HA‐AA][EMIES] and perovskite. (b) FTIR spectra of [HA‐AA][EMIES] and PbI_2_‐[HA‐AA][EMIES]. (c) Pb *4f* XPS spectra of PVK and PVK‐[HA‐AA][EMIES], respectively. (d) A comparison of ^1^H NMR spectra of FA^+^ and FA^+^‐[HA‐AA][EMIES]. The molecular structure of FA^+^ is depicted in the inset of the figure. The H atoms in FA^+^ are marked as ‘a’ and ‘b’, respectively. (e–g) GIWAXS patterns (e), KPFM patterns (f), and contact potential difference (g) of PVK and PVK‐[HA‐AA][EMIES]. The scale bar in (f) is 400 nm.

During perovskite film formation, the volatilization of organic components and the oxidation of halogens inevitably leave behind undercoordinated Pb^2+^ cations at the surface and grain boundaries. These undercoordinated sites are notorious for acting as deep‐level trap states, facilitating non‐radiative carrier recombination and serving as initiation points for moisture‐induced degradation, thereby compromising both efficiency and stability [37, 38]. The molecular structure of [HA‐AA][EMIES] is intentionally designed to address this issue. Its [EtSO_4_]^−^ and –COO^−^ anions possess high binding affinities for Pb^2+^ [15], enabling them to donate electrons and form robust coordination covalent bonds with these undercoordinated sites (Figure 2a). Concurrently, the strongly electronegative fluorine atoms in the HA segment are well‐positioned to form hydrogen bonds with the protonated ammonium groups of formamidinium cations (FA^+^), further anchoring [HA‐AA][EMIES] within perovskite matrix (Figure 2a).

The coordinate interactions between [HA‐AA][EMIES] and Pb^2+^ were proven through Fourier transform infrared (FTIR) spectroscopy and X‐ray photoelectron spectroscopy (XPS) measurements. The FTIR characteristic peak of ─C═O at 1755 cm^−1^ in [HA‐AA][EMIES] shifted to 1713 cm^−1^ after the addition of PbI_2_, and the ─S═O characteristic peak at 1381 cm^−1^ shifted to 1350 cm^−1^ (Figure 2b). The XPS spectral profiles revealed that the binding energies of the Pb 4*f_7/2_* (138.97 eV) and Pb 4*f_5/2_* (143.79 eV) peaks were downshifted to 138.42 and 143.32 eV after [HA‐AA][EMIES] modification, respectively (Figure 2c). These results suggested the formation of coordination between [EtSO_4_]^−^ and Pb^2+^, and between ─COO^−^ and Pb^2+^. Meanwhile, the strongly electronegative F atoms in HA possess a high negative charge density around them, effectively attracting the positively charged H atoms in the ammonium groups of FA^+^ to form hydrogen bonds (Figure 2a). From the ^1^H NMR spectra, the resonance signal of the protonated ammonium in FA^+^ appeared at 7.80 ppm (Figure 2d). With the addition of [HA‐AA][EMIES], the ammonium resonance signal split into two peaks, indicating the altered local environments of ammonium protons. The additive disturbs FA^+^ local symmetry and slows the proton‐exchange kinetics, yielding two well‐resolved resonances on the NMR timescale. This spectral feature verifies the strong intermolecular interactions between FA^+^ and [HA‐AA][EMIES]. This coordination process has a profound impact on the pre‐nucleation state of precursor solution [39]. Dynamic light scattering (DLS) measurements showed that the pristine precursor solution contained small colloids with an average size of 0.70 nm (Figure S7). The addition of HA‐AA increased the colloidal size significantly. Remarkably, the incorporation of [HA‐AA][EMIES] led to a further increase in colloidal size to approximately 150 nm, a 1.5‐fold enhancement over the HA‐AA system. This significant enlargement is attributed to the multifunctional cross‐linking ability of [HA‐AA][EMIES], which aggregates precursor species through the dual action of Pb^2+^ coordination and FA^+^ hydrogen bonding. The formation of these larger and more uniform colloids promotes homogeneous nucleation, which is critical for realizing a high‐quality and defect‐poor film.

The benefits of this controlled precursor chemistry are vividly manifested in the final film morphology and crystallography. From the SEM measurements, PVK‐[HA‐AA][EMIES] held grains that are smoother and more regular compared with PVK and PVK‐HA‐AA, growing perpendicular to the substrate (Figure S8). We conducted synchrotron‐based 2D grazing incidence wide‐angle X‐ray scattering (GIWAXS) measurements to further evaluate the crystallization of these perovskite films. The GIWAXS profiles of PVK, PVK‐HA‐AA, and PVK‐[HA‐AA][EMIES] are depicted in Figure 2e and Figure S9. In PVK, peaks corresponding to PbI_2_ and the yellow phase (*δ*‐phase) were observed at *q*_z_ = 0.88 and 0.85 Å^−1^, respectively. However, the PbI_2_ and *δ*‐phase nearly disappeared in PVK‐[HA‐AA][EMIES], indicating that [HA‐AA][EMIES] significantly promotes the transition from *δ*‐phase to black phase (*α*‐phase) (*q*_z_ = 1 Å^−1^). The X‐ray diffraction (XRD) results also indicated that PVK‐[HA‐AA][EMIES] has higher crystallinity at the position of *α*‐FAPbI_3_ (2*θ* = 14.10°) (Figure S10).

To directly visualize the impact of [HA‐AA][EMIES] on the phase transition kinetics, we performed in situ XRD tracking during annealing process (Figure S11). The wet film (PVK) displayed a complex array of intermediate solvate phases with 2*θ* at 6.56^°^, 7.18^°^, 7.66^°^, and 9.20^°^. It suggested the formation of MA_2_Pb_3_I_8_·2DMSO, indicative of multiple and competing nucleation pathways. The *δ*‐FAPbI_3_ phase persisted throughout the crystallization of *α*‐phase, suggesting an inefficient and incomplete phase transformation [40]. In contrast, the wet film containing [HA‐AA][EMIES] showed a significant suppression of these complex solvate phases. Instead, a new intermediate phase emerged, tentatively assigned as [HA‐AA][EMIES]·*δ*‐FAPbI_3_. Crucially, this intermediate facilitated a direct and rapid transition to pure *α*‐FAPbI_3_ upon annealing, bypassing the convoluted multi‐pathway nucleation and confirming that the ionogel enforces a singular and efficient crystallization route.

The culmination of these synergistic effects, passivation of undercoordinated sites, controlled pre‐nucleation and a direct phase transition, leads to a film with uniform electronic distribution. Kelvin probe force microscopy (KPFM) measurements proved a remarkably uniform surface potential distribution across PVK‐[HA‐AA][EMIES] (Figure 2f,g). This homogeneity indicates a reduction in localized energetic disorder and trap states, which is a direct consequence of the more perfect and well‐passivated crystal structure. By orchestrating the entire film‐forming process from solution to solid, [HA‐AA][EMIES] establishes an ideal foundation for achieving the long‐range carrier diffusion and ultimately, high‐performance PSCs.

### 2.3 Mechanisms of Strain Release and Mechanical Enhancement in Perovskite Films

The residual strain within perovskite lattice is a primary and pervasive source of instability, exacerbating lattice distortions and generating deleterious defective states that undermine both efficiency and long‐term device durability [41, 42]. This strain predominantly originates from the coefficient of thermal expansion (CTE) mismatch between the perovskite film and the underlying substrate during the post‐annealing cooling process [43]. We employed grazing incidence XRD (GIXRD) to quantitatively assess how our designed [HA‐AA][EMIES] mitigates this issue. This technique allows for a sensitive probe of lattice parameter variations as functions of depth and orientation, providing a direct measure of residual strain within perovskite film. We focused the analysis on the (110) crystal plane, selected for its well‐documented stability and reproducibility [44]. By fixing a shallow incident angle (*φ* = 1°) and varying the tilt angle (*ψ*) from 10° to 50°, we could map the strain state within the film.

**FIGURE 3:**
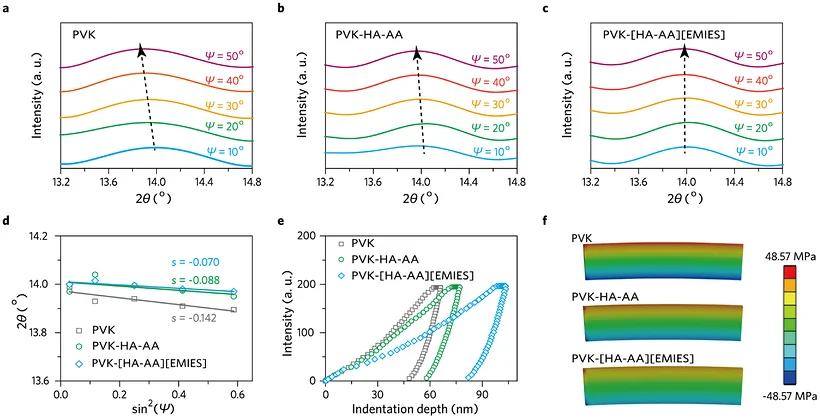
Residual strain and mechanical strain distributions of perovskite films. (a–c) The GIXRD diffraction peaks in the condition of *φ* = 1° shift as instrumental *ψ* value going from 10° to 50° for PVK (a), PVK‐HA‐AA (b), and PVK‐[HA‐AA][EMIES] (c). (d) Dependence of 2*θ* on sin^2^ *ψ* of the corresponding perovskite films and their linear fitting. (e) Loading and unloading force curves obtained from nano‐indentation measurements of PVK, PVK‐HA‐AA and PVK‐[HA‐AA][EMIES]. (f) The finite element analysis of strain distribution in these perovskite films under bending condition. The color scale represents stress in unit of MPa, where the positive and negative values correspond to tensile and compressive stress, respectively.

For PVK, a clear and progressive shift of the (110) diffraction peak was observed to lower 2*θ* angles with increasing *ψ* (Figure 3a). This shift corresponds to an enlargement of the interplanar spacing, *d*_(110)_, which is a hallmark of increasing tensile strain within the lattice. The incorporation of HA‐AA offered the marginal relief, as evidenced by a slightly reduced peak shift (Figure 3b), consistent with its limited mechanical flexibility. In contrast, PVK‐[HA‐AA][EMIES] exhibited a minimal shift of the (110) peak across the entire *ψ* range (Figure 3c), providing the direct crystallographic evidence that [HA‐AA][EMIES] substantially alleviates residual strain. This observation is quantified by plotting 2*θ* as a function of sin^2^ *ψ*, where the linearly fitting slope is proportional to the magnitude of residual strain. PVK‐[HA‐AA][EMIES] displayed the smallest slope, confirming its superior strain‐relief capability (Figure 3d).

To correlate this lattice‐scale strain relief with the macroscopic mechanical behavior of perovskite film, we performed nano‐indentation measurements (Figure 3e and Table S1). We used the Young's modulus (*E*) to evaluate the rigidity of perovskite films. The *E* of PVK‐[HA‐AA][EMIES] was 22.94 GPa, which was significantly lower than those of PVK (37.95 GPa) and PVK‐HA‐AA (27.30 GPa). This substantial reduction in rigidity is a direct consequence of the ionogel's presence at the grain boundaries. The soft, elastomeric [HA‐AA][EMIES] acts as a mechanical buffer, effectively lowering the overall stiffness of the film and enhancing its capacity to accommodate mechanical stress without fracturing. The practical implications of this enhanced mechanical compliance were further explored through finite element method simulations. The simulated stack structure is indium tin oxide (ITO)/ Tin(IV) oxide (SnO_2_)/ perovskite, and the detailed parameters required for simulation are listed in Table S2. The simulation of PVK stack revealed a pronounced concentration of tensile stress at the top surface of perovskite layer and compressive stress at the SnO_2_/PVK interface (Figure 3f).

### 2.4 Carrier Extraction Kinetics of Perovskite Films

The ultimate figure of merit for a photovoltaic absorber is its ability to efficiently transport the photogenerated carriers over long distances without recombination. Having established that [HA‐AA][EMIES] orchestrates superior film crystallization and effectively releases residual strain, we sought to directly visualize and quantify the impact of these synergistic effects on the fundamental carrier transport dynamics. To this end, we employed a powerful combination of transient absorption (TA) spectroscopy and transient absorption microscopy (TAM) [45]. TAM is uniquely suited for this investigation, as it enables the direct, real‐time visualization of carrier migration in both spatial and temporal dimensions, providing a direct window into long‐range transport phenomena within the polycrystalline film [46, 47].

We first examined the carrier recombination kinetics using TA spectroscopy under a moderate excitation fluence of 0.48 µJ cm^−^^2^ (corresponding to a carrier density of ∼2.4 × 10^1^ ^6^ cm^−^^3^). The TA spectra for all films (PVK, PVK‐HA‐AA, and PVK‐[HA‐AA][EMIES]) exhibited a prominent ground‐state bleaching (GSB) feature centered around 767 nm, which evolved with delay time (Figure S12a–c). By tracking the recovery of this GSB signal, we probed the carrier recombination dynamics. As shown in Figure S12d, the decay kinetics at 767 nm reveal a stark contrast between the samples. PVK‐[HA‐AA][EMIES] exhibited a remarkably slower recombination rate compared to both PVK and PVK‐HA‐AA, providing an initial indication of substantially suppressed non‐radiative loss.

**FIGURE 4:**
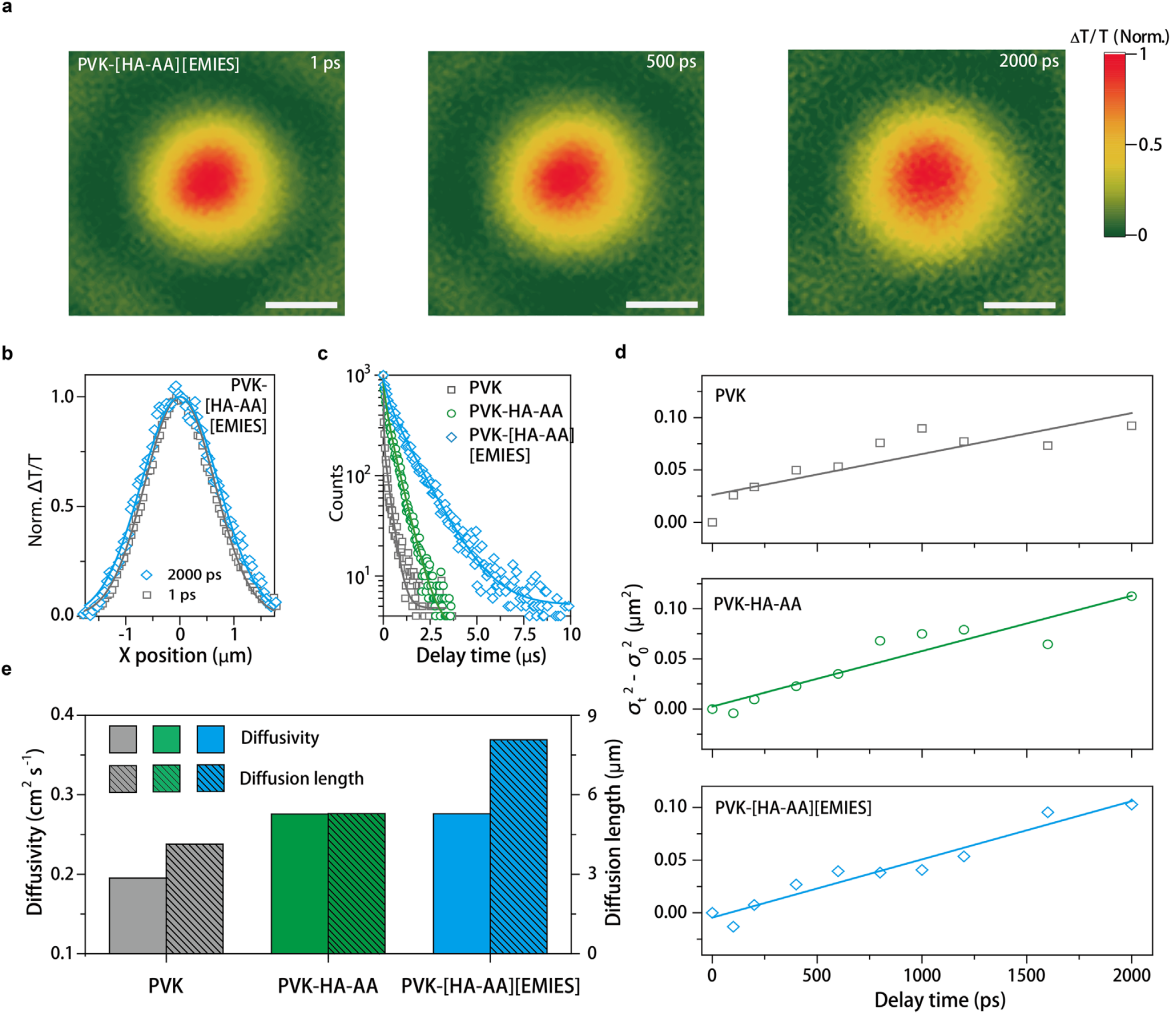
Carrier extraction kinetics of PVK, PVK‐HA‐AA and PVK‐[HA‐AA][EMIES]. (a) 2D TAM imaging of PVK‐[HA‐AA][EMIES] at different delay time under a pump fluence of 1.05 µJ cm^−2^. The color scale represents the intensity of pump‐induced ΔT of the probe, and every image has been normalized by peak value. The scale bar is 1 µm. The probe wavelength is 767 nm. (b) Scans of excited‐state density profile projected along one dimension of PVK‐[HA‐AA][EMIES]. (c) TRPL spectra of PVK, PVK‐HA‐AA and PVK‐[HA‐AA][EMIES]. (d) Diffusivity of PVK, PVK‐HA‐AA and PVK‐[HA‐AA][EMIES] obtained through fitting the variances of Gaussian profiles. The data are averaged from three independent samples. (e) The diffusivity values and their corresponding diffusion lengths for PVK, PVK‐HA‐AA and PVK‐[HA‐AA][EMIES].

The pseudo‐color TAM images in Figure 4a and Figure S13 captured the spatial evolution of photogenerated carrier population over time. At initial delay times, all films showed a positive GSB signal with a well‐defined Gaussian distribution. As time progresses, this distribution broadened isotropically, providing a direct visualization of carrier diffusion away from the generation point. This isotropic behavior allows us to condense the analysis to one dimension without loss of information, thereby achieving higher resolution for rigorous kinetic modeling. The 1D carrier density profiles at successive delay time are perfectly described by a Gaussian function (Figure 4b), and the time‐dependent broadening of these profiles is a direct measure of the diffusion coefficient.

Complementary time‐resolved photoluminescence (TRPL) measurements, probing the emission around 820 nm (Figure S14), provided the effective carrier lifetime (*τ*) for each film. The extracted lifetime are summarized in Figure 4c and Table S3. PVK‐[HA‐AA][EMIES] displayed an exceptional average carrier lifetime (*τ*_avg_) of 1.11 µs, a dramatic increase compared to PVK (0.23 µs) and PVK‐HA‐AA (0.49 µs). This significant prolongation of *τ*_avg_ is a direct consequence of the reduced non‐radiative recombination pathways, stemming from the effective defect passivation and superior crystal quality imparted by [HA‐AA][EMIES].

By plotting the variance (*σ* ^2^) of the Gaussian carrier distribution as function of delay time (Figure 4d), we extracted the diffusivity from the linear fit of its time evolution (Figure 4e and Table S3). PVK‐[HA‐AA][EMIES] exhibited the highest diffusivity (*D*), reaching 0.28 cm^2^ s^−^^1^. With both *τ*_avg_ and diffusion constant, we further calculated carrier diffusion length (*L*), a comprehensive metric of transport quality. The results are striking, as PVK‐[HA‐AA][EMIES] achieving an exceptionally long carrier diffusion length of 8.07 µm. This value far surpasses those of PVK (4.13 µm) and PVK‐HA‐AA (5.28 µm). More importantly, it substantially exceeds the current benchmark of ∼1.4 µm reported for state‐of‐the‐art FAPbI_3_‐based polycrystalline films, underscoring the transformative nature of our approach [48].

This comprehensive set of transport measurements provides compelling evidence that [HA‐AA][EMIES] ionogel delivers on its multifunctional design. By simultaneously passivating defects, promoting a more perfect crystal lattice, and releasing residual strain, it drastically reduces the density of trap states that would otherwise scatter carriers and promote recombination [49]. This establishes an ideal foundation for fabricating solar cells with not only high power conversion efficiencies but also the robust charge extraction necessary for operation in next‐generation rigid and flexible devices.

### 2.5 Optoelectronic Performance and Operational Stability of PSCs

**FIGURE 5:**
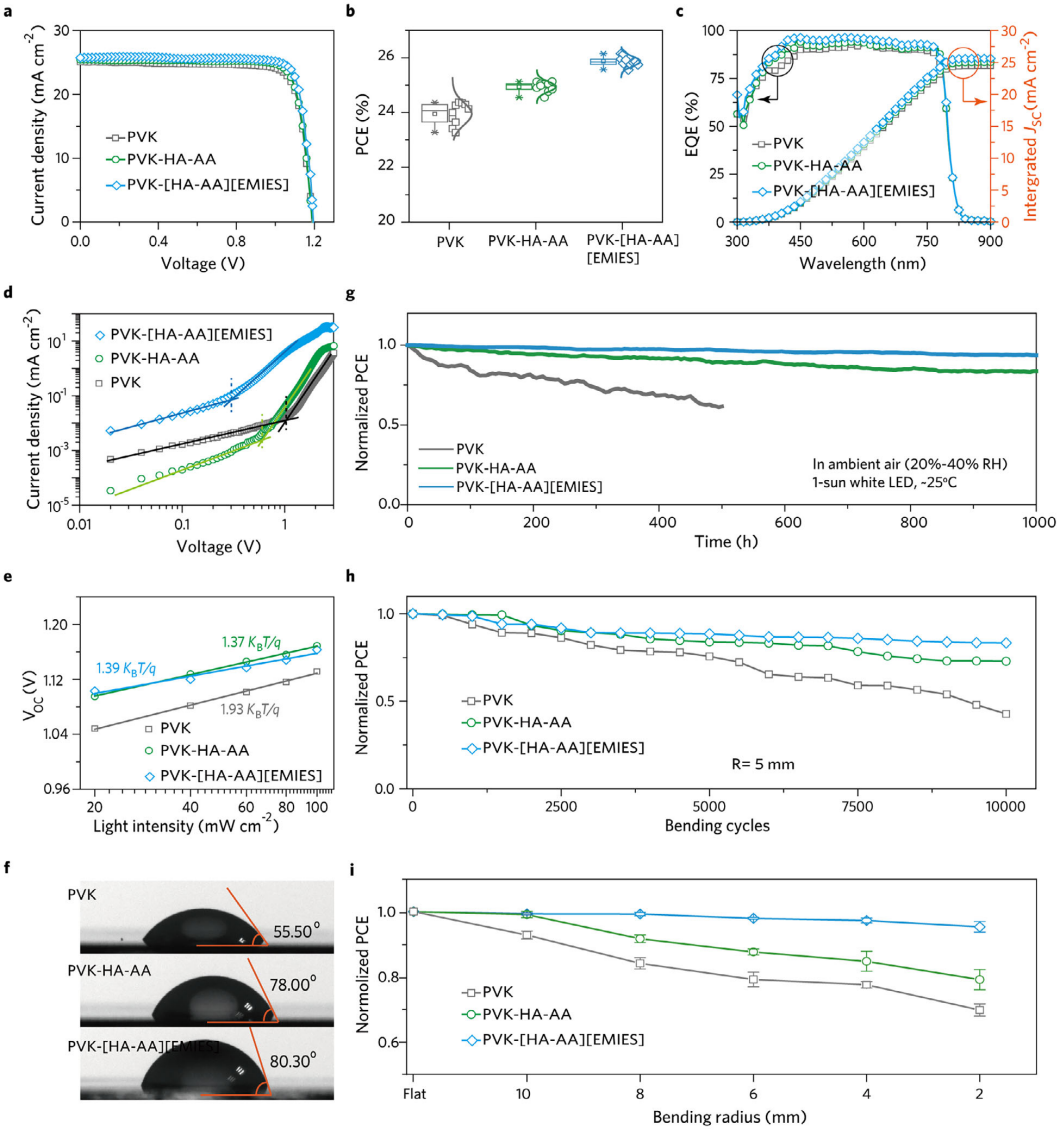
Performance of the photovoltaic devices. (a) *J–V* curves of the champion devices based on PVK, PVK‐HA‐AA and PVK‐[HA‐AA][EMIES]. (b) Efficiency statistics of PSCs, evaluated across ten devices for each condition. (c) EQE and the integrated *J*_SC_ of the PSCs based on PVK and PVK‐[HA‐AA][EMIES]. (d) SCLC measurements of perovskite films. (e) *V*_OC_ as a function of light intensities of PSCs. We fitted the data points with a slope of *nk_B_T/q*, where *n* is the ideality factor, *k_B_* is the Boltzmann constant, *T* is temperature and *q* is the electron charge. (f) Water contact angle images of these perovskite films. (g) Operational stability of encapsulated devices under continuous 1‐sun‐equivalent white‐light LED illumination with MPPT in ambient air. (h) Stability test of the unencapsulated *f*‐PSCs versus bending cycles at a radius of 5 mm. (i) Normalized PCE of *f*‐PSCs after bending at different curvature radius for 1000 cycles. Error bars represent the standard deviation from ten independent devices.

We translated the exceptional bulk properties of FAPbI_3_, including the superior film crystallization, effectively released residual strain, and significantly enhanced carrier transport orchestrated by [HA‐AA][EMIES], into functional devices. We fabricated both rigid and flexible n‐i‐p structured devices incorporating the optimized additive concentration of 2.0 wt.% relative to PbI_2_ (Figure S15). The current density‐voltage (*J–V*) curves of the champion devices are presented in Figure 5a. The PVK‐[HA‐AA][EMIES]‐based device achieved a remarkable PCE of 26.45%, a substantial improvement over the PVK‐based device (24.83%). This enhancement is primarily driven by gains in both fill factor (FF) and open‐circuit voltage (*V*_OC_) (Table S4), directly reflecting the reduced non‐radiative recombination and more efficient charge extraction enabled by the multifunctional passivation and strain‐relief mechanisms of [HA‐AA][EMIES]. The statistical distribution of photovoltaic parameters across ten devices confirmed the excellent reproducibility, with the [HA‐AA][EMIES]‐modified devices yielding the highest average PCE of 25.88%, compared to 24.96% for PVK‐HA‐AA and 23.87% for PVK (Figure 5b).

Critically, the benefits of strain release are amplified in flexible photovoltaics. When incorporated into *f*‐PSCs, [HA‐AA][EMIES] delivered a similarly impressive performance enhancement, boosting the champion PCE from 23.40% in the device based on PVK to 25.14% (Figure S16 and Table S5). It demonstrates that the soft and compliant buffer points at the GBs dedicated by [HA‐AA][EMIES] is particularly advantageous for flexible device architectures.

To deconvolute the origins of these performance gains, we conducted a series of optoelectronic characterizations. External quantum efficiency (EQE) measurements yielded an integrated current density (*J*_SC_) of 25.63 mA cm^−^^2^ for the champion PVK‐[HA‐AA][EMIES]‐based device, which closely aligns with the value obtained from the *J–V* scan under standard AM 1.5G illumination (26.12 mA cm^−^^2^), confirming the accuracy of our efficiency measurements (Figure 5c). Space‐charge‐limited current (SCLC) measurements were employed to quantify the trap density within the devices. As shown in Figure 5d and Table S6, the incorporation of [HA‐AA][EMIES] led to a dramatic reduction in the defect density, from 4.42 × 10^1^ ^5^ cm^−^^3^ in the PVK‐based device to just 1.32 × 10^1^ ^5^ cm^−^^3^. This more than three‐fold reduction provides direct evidence of the ionogel's exceptional defect‐passivating capability. This finding is further corroborated by the measurement of light intensity dependence of *V*_OC_ (Figure 5e). The [HA‐AA][EMIES]‐modified device exhibited a significantly reduced slope of 1.39 *k_B_T/q*, compared to 1.93 *k_B_T/q* for the PVK‐based device. As an ideality factor (*n*) approaching 1, the more suppressed trap‐assisted Shockley‐Read‐Hall recombination is achieved. The result confirms that [HA‐AA][EMIES] effectively pacifies the deep‐level traps and improves *V*_OC_.

The hydrophobicity imparted by the fluorine‐rich components of [HA‐AA][EMIES] was evident from water contact angle measurements. It increased from 55.50° (PVK) to 78.00° (PVK‐HA‐AA) and 80.30° (PVK‐[HA‐AA][EMIES]) (Figure 5f). This enhanced surface barrier against moisture ingress translated directly into superior device durability. Under continuous 1 sun equivalent white light LED illumination with maximum power point tracking (MPPT) in ambient air (∼25°C, 20%–40% RH), the PVK, PVK HA AA, and PVK [HA AA][EMIES] devices decreased to 95% of their initial PCEs after approximately 20, 180, and 810 h, respectively (Figure 5g). The PCE for PVK [HA AA][EMIES] device retains more than 90% over 1,000 h, demonstrating the effectiveness of ionogel network in suppressing photoinduced degradation and maintaining long term operational stability.

For *f*‐PSCs, mechanical stability under repeated bending is a critical benchmark. We therefore subjected our flexible devices to rigorous bending tests. The PVK‐[HA‐AA][EMIES]‐based *f*‐PSC demonstrated the remarkable mechanical endurance, retaining 83% after 10,000 bending cycles in ambient air (bending radius: 5 mm, ∼25°C, 20%–40% RH) (Figure 5h). Under the same conditions, the PVK‐based *f*‐PSC degraded by more than 50%. After 10,000 bending cycles at a radius of 5 mm, cracks in PVK‐[HA‐AA][EMIES] progressively narrowed and were substantially healed within 2 h at room temperature, unlike PVK and PVK‐HA‐AA (Figure S17). This confirms the crack‐recovery capability of the modified perovskite film. The outstanding mechanical robustness originates partly from the self‐healing capability of [HA‐AA][EMIES], which can dynamically repair microcracks at GBs under mild ambient condition. This crack‐healing behavior effectively prevents crack propagation during repeated bending, thus preserving device integrity and photovoltaic performance. This superior flexibility was further validated by bending radius tests (Figure 5i). As the bending radius decreasing to 2 mm, the PVK‐based device suffered a significant PCE drop to 69% after 1,000 bending cycles. In contrast, the PVK‐[HA‐AA][EMIES] based *f*‐PSC maintained 95% of its initial PCE, showcasing an extraordinary tolerance to mechanical stress.

These results collectively underscore the holistic benefits of [HA‐AA][EMIES]. By simultaneously enhancing crystallinity, passivating electronic defects, and introducing a compliant, stress‐relieving network at the GBs, it addresses the primary degradation pathways, both environmental and mechanical, that plague perovskite devices. The consequence is a photovoltaic platform that not only achieves a new level of PCE but also demonstrates the robust atmospheric and mechanical stability required for real‐world deployment, particularly in the burgeoning field of flexible and portable electronics.

## 3 Conclusion

In summary, we have introduced a rationally designed ionogel as a multifunctional additive to address the intertwined challenges of defect passivation and residual strain management in PSCs, thereby unlocking long‐range carrier diffusion. Our approach establishes a dual cross‐linked network in‐situ that orchestrates the entire film‐forming process. This strategy not only suppresses the formation of detrimental intermediate phases during FAPbI_3_ crystallization but also facilitates a direct and efficient transition from the non‐photoactive *δ*‐phase to the desired *α*‐phase. Critically, the ionogel integrates at the grain boundaries, forming dynamic, and ligament‐like structures. These supramolecular networks serve a dual mechanical purpose: they effectively dissipate residual lattice strain that would otherwise degrade performance, while simultaneously imparting enhanced toughness and autonomous self‐healing capability to the perovskite film. This synergistic effect, simultaneously addressing electronic passivation and mechanical relaxation, leads to a transformative improvement in both the PCE and operational stability of PSCs. The resulting devices achieve champion PCEs of 26.45% for rigid PSCs and 25.14% for *f*‐PSCs, with negligible degradation observed during rigorous long‐term aging and mechanical bending tests. Our findings establish ionogel engineering as a powerful and versatile strategy that addresses key commercialization barriers, suggesting that such materials will play an increasingly vital role in the future of stable and high‐performance perovskite photovoltaics.

## Author Contributions

**Wen Wu**: methodology, data curation, investigation, formal analysis, writing – original draft. **Gang Wang**: methodology, data curation, investigation, formal analysis, writing – original draft. **Chen Liu**: methodology, data curation, investigation, formal analysis, writing – original draft. **Xiao‐Li Du**: methodology, data curation, investigation, formal analysis, writing – original draft. **Sheng Yang**: methodology, data curation, investigation, formal analysis, writing – original draft. **Shao‐Long Wang**: methodology, data curation, investigation, formal analysis. **Jia‐Yi Tian**: methodology, data curation, formal analysis, investigation. **Ling‐Feng Chao**: methodology, data curation, investigation, formal analysis. **Xiang‐Feng Deng**: methodology, data curation, investigation, formal analysis, writing – original draft. **Yi Jiang**: methodology, data curation, investigation, formal analysis, supervision, writing – original draft, writing – review and editing. **Yong‐Hua Chen**: methodology, formal analysis, supervision, resources, writing – review and editing. **Gui‐Chuan Xing**: methodology, formal analysis, supervision, resources, writing – review and editing. **Wen‐Yong Lai**: conceptualization, methodology, formal analysis, funding acquisition, project administration, resources, writing – review and editing.

## Conflicts of Interest

The authors declare no conflicts of interest.

## Supporting information




**Supporting File**: advs77804‐sup‐0001‐SuppMat.pdf.

## Data Availability

The data that support the findings of this study are available in the Supporting Information of this article.
