# Supplementary material for: Self‐Healing Ionogel Dual‐Network Enables Synergistic Defect Passivation and Strain Relief for High‐Efficiency Robust Perovskite Photovoltaics
Source: Adv Sci (Weinh). 2026 Sep 27:e77804. Online ahead of print. doi: 10.1002/advs.77804 (PMC13616255; doi:10.1002/advs.77804)
Supplement: Supplementary file 1 [file ADVS-9999-e77804-s001.pdf]

Supplementary Information

Self-healing Ionogel Dual-network Enables Synergistic Defect Passivation and Strain Relief for High-efficiency Robust Perovskite Photovoltaics

Wen Wu,[a] Gang Wang,[b] Chen Liu,[a] Xiao-Li Du,[a] Sheng Yang,[a] Shao-Long Wang,[a] Jia-Yi Tian,[a] Ling-Feng Chao,[c] Xiang-Feng Deng,[b] Yi Jiang,*[a] Yong-Hua Chen,[c] Gui-Chuan Xing,[b] and Wen-Yong Lai*[a]

Wen Wu and Gang Wang contributed equally to this work.

[a] W. Wu, C. Liu, X. Du, S. Yang, S. Wang, J. Tian, Prof. Y. Jiang,* and Prof. W.-Y. Lai*
State Key Laboratory of Flexible Electronics (LoFE), Institute of Advanced Materials (IAM), School of Chemistry and Life Sciences
Nanjing University of Posts & Telecommunications
9 Wenyuan Road, Nanjing 210023, China
E-mail: iamyjiang@njupt.edu.cn; iamwylai@njupt.edu.cn

[b] G. Wang, X. Deng, and Prof. G. Xing
Joint Key Laboratory of the Ministry of Education, Institute of Applied Physics and Materials Engineering
University of Macau
Taipa, Macao SAR 999078, China

[c] Prof. L. Chao, and Prof. Y. Chen
State Key Laboratory of Flexible Electronics (LoFE) and Institute of Advanced Materials (IAM), School of Flexible Electronics (Future Technologies)
Nanjing Tech University
5 Xinmofan Road, Nanjing 210009, China

*E-mail: iamyjiang@njupt.edu.cn (Y. Jiang); iamwylai@njupt.edu.cn (W.-Y. Lai).

Experimental Section/Methods

Synthesis and characterization of HA-AA and [HA-AA][EMIES]: All materials and solvents were used as received without further purification. HA (98%), AA (98%), [EMIES] (≥95%) and 2-hydroxy-4'-(2-hydroxyethoxy)-2-methyl-propiophenone (98%) were purchased from Sigma-Aldrich.

For the synthesis of HA-AA, HA and AA were mixed in a prescribed molar ratio of 1:1, with 2-hydroxy-4'-(2-hydroxyethoxy)-2-methyl-propiophenone as the photoinitiator at 1% of the total molar amount of the monomers. The aforementioned mixed precursors were stirred at room temperature for 30 minutes until they became clear and well-mixed. Subsequently, the solution was sonicated for 10 minutes to eliminate any bubbles. Two glass plates were clamped together with two release films in between, and a 1.5 mm silicone spacer with a small cutout was placed in the middle. The uniform mixed solution was then injected into the silicone spacer. The edges of the mold were clamped with a dovetail clip to form a sealed space to isolate oxygen, and the mold was then placed in a UV curing apparatus for photopolymerization for 6 hours to obtain the sample. The same preparation method was used for [HA-AA][EMIES], except that 20 wt% [EMIES] was added to the mixed solution.

For self-healing measurements, rhodamine 6G and methyl blue were added separately to stain the samples as red and blue, respectively, during the preparation of HA-AA and [HA-AA][EMIES]samples. The samples of both colors were compression molded using a dog-bone-shaped mold and subsequently cut into two halves. The cut surfaces of the red and blue fragments were then brought together and healed at 60°C and room temperature for 1 hour, respectively.

^{1}H NMR measurements were conducted in d_6-DMSO using a Bruker AVANCE NEO 400 MHz instrument. FTIR spectra were recorded on a PerkinElmer spectrometer spectrum Two. HA-AA and [HA-AA][EMIES] were separately subjected to freeze-drying and then grinded into powders. The powders were blended with potassium bromide, pressed into tablets, and subjected to testing. TGA

measurement was conducted in a nitrogen atmosphere using a thermogravimetric analyzer (NETZSCH STA2500). The temperature was increased at a heating rate of 10 °C/min . The tensile property was measured using a tensile-compressive tester (Instron 5944) with a stretched deformation rate of 5 mm min^{-1}.

Fabrication and characterization of perovskite films: *N,N*-dimethylformamide (DMF, 99.8% purity) and DMSO (anhydrous, ≥99.9%) were purchased from Sigma-Aldrich. Formamidinium iodide (FAI, >99.5%) and MACl (>99.5%) were purchased from GreatCell Solar Materials. PbI_2 (99.99%) was purchased from TCI Chemicals. The perovskite precursor of 1.54 M $FAPbI_3$ was prepared by dissolving 264.83 mg FAI, 709.96 mg PbI_2, and 33.76 mg MACl in the 1 mL DMF/DMSO (4:1, v/v) mixed solvent. For the PVK-HA-AA and PVK-[HA-AA][EMIES] precursor solution, HA-AA and [HA-AA][EMIES] were dissolved in the precursor solution, respectively. On the FTO substrate, perovskite precursor solutions were spin-coated respectively, the process of 1000 rpm for 10 seconds was first performed, and followed by a second spin-coating step at 5000 rpm for 30 seconds. Diethyl ether (1000 μL) was dropped onto the spin-coated substrate at 20 seconds. After spinning, the substrates were immediately annealed at 150°C for 10 minutes to form perovskite films.

FTIR, IR and ^{1}H NMR spectra were recorded by using the same equipment as mentioned for perovskite precursor and samples. SEM measurements were conducted on Hitachi S4800 scanning electron microscope and operated at an acceleration voltage of 8 kV. For HR-TEM characterization, the perovskite films were gently scraped from the substrate and dispersed in chlorobenzene (CB) by mild agitation. A small aliquot of the resulting dispersion was drop-cast onto lacey carbon-coated Cu grids and dried completely before characterization. HR-TEM measurements were performed using a Thermo Fisher Scientific Talos F200X microscope. AFM images were measured using a Dimension FastScan (Bruker) atomic force microscope. A D8 Advance A25 (Bruker) X-ray diffractometer with

Bragg-Brentano para focusing geometry was employed to test XRD patterns. GIWAXS measurements were conducted at the BL14B1 beamline of the Shanghai Synchrotron Radiation Facility. The incident angle was set at 0.40°. The GIWAXS data were analyzed using Fit-2D and presented in q coordinates. PL and TRPL spectra were obtained by FLS1000 with an excitation wavelength of 540 nm. XPS measurements were conducted using the Axis Supra (SHIMADZU) scanning XPS microprobe with a monochromatic Al Kα X-ray source of 24.8 watts and a beam size of 100 μm, at a pressure of 5.0×10^{-7} Pa. To correct for charge shifts in binding energies, all peaks were calibrated with respect to the C *1s* peak located at 284.8 eV. KPFM measurements were performed using a SCM-PIT-V2 probe with the Dimension FastScan AFM system (Bruker Corporation). Contact angle measurements were performed using a KRUSS DSA20. Samples were placed on the sample stage for positional leveling calibration. An electric injection was used to dispense droplets, and image acquisition was performed once the sample had come into stable contact with the droplets. DLS measurements were conducted on a Brookhaven ZetaPALS equipped with a 10 mW HeNe laser at a wavelength of 633 nm. The precursor solution was repeatedly filtered through a 0.22 μm organic-system filter membrane. The filtered solution was then slowly injected into the test cell to avoid bubble formation. After placing the sample in the device, testing commenced following a 2-minute stabilization period.

GIXRD measurements were performed using a Rigaku SmartLab five-axis X-ray diffractometer at 45 kV and 200 mA with Cu Kα radiation (λ = 1.54050 Å). Nano-indentation measurements were conducted on a Bruker Hysitron TI980. The indenter type utilized was a Vickers indenter, composed of diamond material and shaped as a triangular pyramid. The tip curvature radius was 30 nm. A quasi-static trapezoidal testing method was employed, with loading, holding and unloading durations of 5,

2 and 5 seconds, respectively. The maximum load applied during the test was 200 μN, and the loading and unloading rate were 40 μN/s. E was calculated by the following equation:

$$E = \frac{S\sqrt{\pi}}{2\sqrt{A}},$$

where S and A represent the slope of the unloading curve and the contact area between indenter and perovskite film.

To investigate the mechanical reliability of perovskite films, we employed finite element analysis to simulate the strain distribution within perovskite films under bending conditions. E of different perovskite films were obtained through nano-indentation measurement. The thickness information for all layers was listed in Supplementary Table 2. After constructing the model, it was imported into the solution module for material assignment and mesh generation, with a mesh element size of 0.01 μm. Subsequently, a load of 1 μN was applied, causing tensile strain on the top two sides of the model and compressive stress on the bottom two sides. Equivalent elastic strain, equivalent stress and normal strain were added in the solution options. By solving the equations, we simulated and calculated the three different strain distributions within perovskite films under bending conditions. The finite element analysis of strain distribution was performed by ANSYS Workbench 2023 R1.

Transient spectroscopy of perovskite films: An Astrella F Ti:sapphire amplifier (Coherent) pumped by a Revolution-50 (Coherent) nanosecond laser, was used as a light source (output at 800 nm, 1 kHz repetition rate). The high energy (~7 mJ/pulse) and ultrashort (~100 fs pulse duration) femtosecond laser is guided into OPerA Solo optical parametric amplifier (Coherent OPA) to obtain broadband and wavelength-tunable laser. The output laser was then guided to different optical path for TAM and TA spectroscopy.

In the TAM optical path, 50% of the induced pulse energy was guided into OPA to generate probe light with the desired wavelength, while the remainder 50% pulse energy was doubled to 400

nm and serves as a pump beam. The repetition rate was 1 kHz for both beams. A 100×/NA=0.80 objective (Olympus LMPLANFL N) was firstly used to focus the laser beam onto the sample, and the transmitted light was then collected by another objective (100×/NA=0.80) and detected by a CMOS (Andor, Zyla 42). The probe beam and pump beams were aligned concentrically, with probe beam five times larger than pump beam to achieve wide-field detection. Between the two objective lenses, a sample stage was positioned with three-dimensional freedom of movement and equipped with a pair of clamps that firmly hold the sample in place. To collect charge diffusion imaging, the optical path length of pump beam was kept constant, while the probe beam's arrival time to the sample—relative to the pump beam—was controlled by varying its path length with a delay stage (Thorlabs).

The pump pulse for TA system was generated by the same laser system. The broadband white light was produced by focusing the low-power 800 nm femtosecond fundamental pulses into a piece of sapphire crystal. A chopper blocked every other pump pulse, giving the pump beam an effective repetition rate of 500 Hz, while the probe beam remained at 1 kHz repetition rate. Both the pump and probe beams were focused onto the same point on the sample, with the pump beam broader than the probe beam to ensure uniform excitation. Time delay was adjusted by delaying the pump pulse optically using a mechanical delay stage. After transmitting through the sample, the probe light was then collected by detector using optical fibers connected to spectrometers.

Fabrication and performance of PSCs and f-PSCs: CB (99.8% purity), lithium bis(trifluoromethanesulfonyl) imide (Li-TFSI, ≥99%), 4-tert-butyl-pyridine (TBP, >96.0%) and acetonitrile (ACN, 99.8%, anhydrous) were purchased from Sigma-Aldrich. SnO_2 colloidal dispersion (15% in H_2O) was purchased from Xi'an Polymer Light Technology Corp. *F*-doped tin oxide (FTO) glass (8 Ω sq^{-1}), polyethylene glycol terephthalate/indium-tin oxide (PET/ITO) (15 Ω

sq^{-1}) and 2,2',7,7'-tetrakis(*N*,*N*-diphenylamino)-9,9'-spirobifluorene (Spiro-OMeTAD, >99.8%) were purchased from Advanced Election Technology Co., Ltd. Glass/FTO and PET/ITO were cleaned in an ultrasonic bath in the sequence of washing solution, deionized water, acetone and ethanol, with each step lasting for 15 minutes. Then the substrates were treated with UV-ozone for 20 minutes to improve hydrophilicity. For the deposition of SnO_2 layer, the SnO_2 aqueous solution was diluted with deionized water (volume ratio of 1:4) and dispersed ultrasonically for 30 minutes. The SnO_2 solution was then spin-coated on FTO at a speed of 4000 rpm for 30 seconds and annealed at 150°C for 30 minutes. Subsequently, the perovskite film was spin-coated onto SnO_2. The hole-transport solution was prepared by dissolving 28.8 µL of TBP, 17.6 µL of Li-TFSI solution (520 mg in 1 mL ACN) and 73.2 mg of Spiro-OMeTAD in 1 mL CB. Then spin-coated on perovskite films by 2500 rpm for 30 seconds. Finally, a 10 nm thick MoO_3 (at a rate of 0.2 Å s^{-1}) and 100 nm thick Ag (at a rate of 2.0 Å s^{-1}) anode were deposited by thermal evaporation to complete the preparation of PSCs with an effective area of 0.05 cm^2. The PSCs were fabricated with a conventional structure of glass/FTO/SnO_2/perovskite/Spiro-OMeTAD/MoO_3/Ag. The PET/ITO substrates were used for *f*-PSCs instead of rigid glass/FTO substrates.

J-V, SCLC and the light intensity-dependent V_{OC} measurements were conducted by using a 2400 Series Source Meter (Keithley Instruments) under a SS-F5-3A solar simulator (AM 1.5G illumination, 100 mW cm^{-2}) (Enlitech) calibrated by a NREL standard Si cell, and no additional UV filter equipment was used. *J-V* measurements were carried out with the devices inside the glove box (<0.1 ppm O_2 and H_2O). To ensure the accuracy of J_{SC} from *J-V* scans, a mask with an aperture area of 0.05 cm^2 was covered during the measurement. The *J-V* curves were scanned in reverse (forward bias (1.3 V) → short circuit (-0.2 V)) or forward (short circuit (-0.2 V) → forward bias (1.3 V)) with a scan rate of 50 mV/s, and 1 ms delay time. EQE measurements were made on a QTest Station 2000 ADI

system (Crowntech, Inc.). SCLC measurements were performed on an electron-only device, i.e., FTO/SnO_2/ perovskite/PCBM/Ag. The Au layer was deposited by thermal evaporation. The trap densities (N_t) of perovskite films were calculated using the following equation: $N_t = \frac{2V_{TFL}\varepsilon_0\varepsilon_r}{eL^2}$, where e, L, ε_0 and ε_r represent the electron charge, the thickness of the perovskite film, vacuum permittivity and relative dielectric constant of perovskite, respectively. For light intensity-dependent V_{OC} measurements, V_{OC} was recorded sequentially by setting the light intensity from 20 to 100 mW cm^{-2} in steps of 20 mW cm^{-2}.

For mechanical stability measurements, the devices were stored in a temperature- and humidity-controlled cabinet with a transparent glass door (~25°C and 20-40% RH). The bending durability of *f*-PSCs was evaluated using a mechanical tester (PR-BDM-100, Puri Materials) in the constant-radius bending mode in the air glove box. The tests of the unencapsulated *f*-PSCs versus bending cycles at a radius of 5 mm, conducted in the dark under ambient air at ~25°C and 20-40% RH. The tests of unencapsulated *f*-PSCs after bending at different curvature radii for 1,000 cycles. The bending tests were conducted in the dark under ambient air at ~25°C and 20-40% RH. Operational stability of encapsulated devices was performed under continuous 1-sun-equivalent white-light LED illumination with MPPT in ambient air at ~25°C and 20-40% RH.

The calculation of carrier diffusion length *L*

The charge diffusion process in perovskite films in absence of an electric field is modelled as in-plane 2D diffusion because the signal is integrated over out-of-plane direction. The pump intensity is kept at the same level as that of TA measurement. Hence, the GSB intensity is in proportion to the carrier density. As time evolves, the carrier distribution profile broadens_gradually, which indicates an isotropic carrier diffusion. Considering the carrier diffusion and recombination, the time-dependent carrier distribution can be given by:

$$\frac{\partial n(x,y,t)}{\partial t} = D\left[\frac{\partial^2 n(x,y,t)}{\partial x^2} + \frac{\partial^2 n(x,y,t)}{\partial y^2}\right] - \frac{n(x,y,t)}{\tau} - \gamma n^2(x,y,t) - \eta n^3(x,y,t),$$

where $n(x,y,t)$ is the carrier population as a function of position and time, D is the diffusivity, τ is the carrier lifetime, γ is the bimolecular recombination coefficient and η is the Auger recombination coefficient. The initial population $n(x,y,0)$ follows a 2D Gaussian distribution as created by a Gaussian pump beam at position (x_0, y_0):

$$n(x,y,0) = Nexp\left[-\frac{(x-x_0)^2}{2\sigma_{0,x}^2} - \frac{(y-y_0)^2}{2\sigma_{0,y}^2}\right],$$

$$n(x,y,t) = Nexp\left[-\frac{(x-x_0)^2}{2\sigma_{t,x}^2} - \frac{(y-y_0)^2}{2\sigma_{t,y}^2}\right].$$

At low excitation intensity where the higher order recombination terms are negligible, the solution of equation (1) reduces into a 2D Gaussian distribution representing a spreading out population as a function of delay time *t*. For an isotropic diffusion, we can focus on the carrier diffusion along one direction (such as x axis) to reflect the holistic carrier diffusion,

$$\sigma_{t,x}^2 = \sigma_{0,x}^2 + L^2 = \sigma_{0,x}^2 + 2Dt,$$

where $\sigma_{t,x}^2$ describes the variance at time *t*, $\sigma_{0,x}^2$ is the variance of the initial distribution created by the laser pulse, and *D* is the diffusivity.

D was calculated by linear fitting of the time evolution of Gaussian variance of $\sigma^2(t)$-$\sigma^2(0) = 2Dt$, where $\sigma^2(0)$ represent the initial variance after excitation.

The calculation of light intensity-dependent V_{OC} measurements

The relationship between response photovoltaic parameter (V_{OC}) and applied light intensity (*I*) can be expressed by the following formula:

$$V_{OC} = \frac{nk_BTln\left(\frac{J_{SC}}{J_0}\right)}{q} = \frac{\alpha nk_BTln(I)}{q} + c,$$

where α is an empirical parameter, n is ideal factor, k_B is Boltzmann constant, J_0 is the reverse saturation current density, q is elementary charge, T is temperature and c is a fitting parameter.

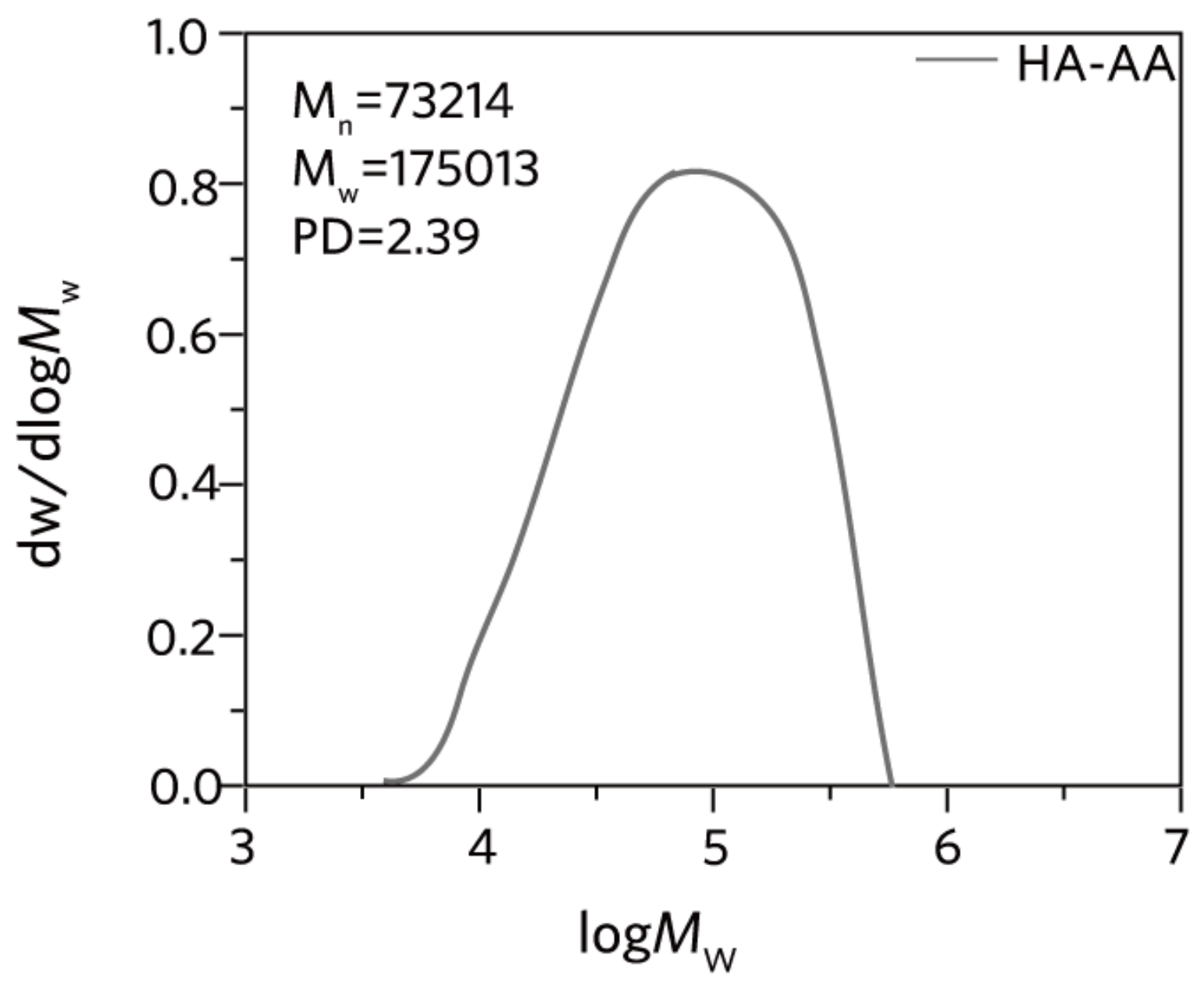

Figure S1. The gel permeation chromatography curve of HA-AA.

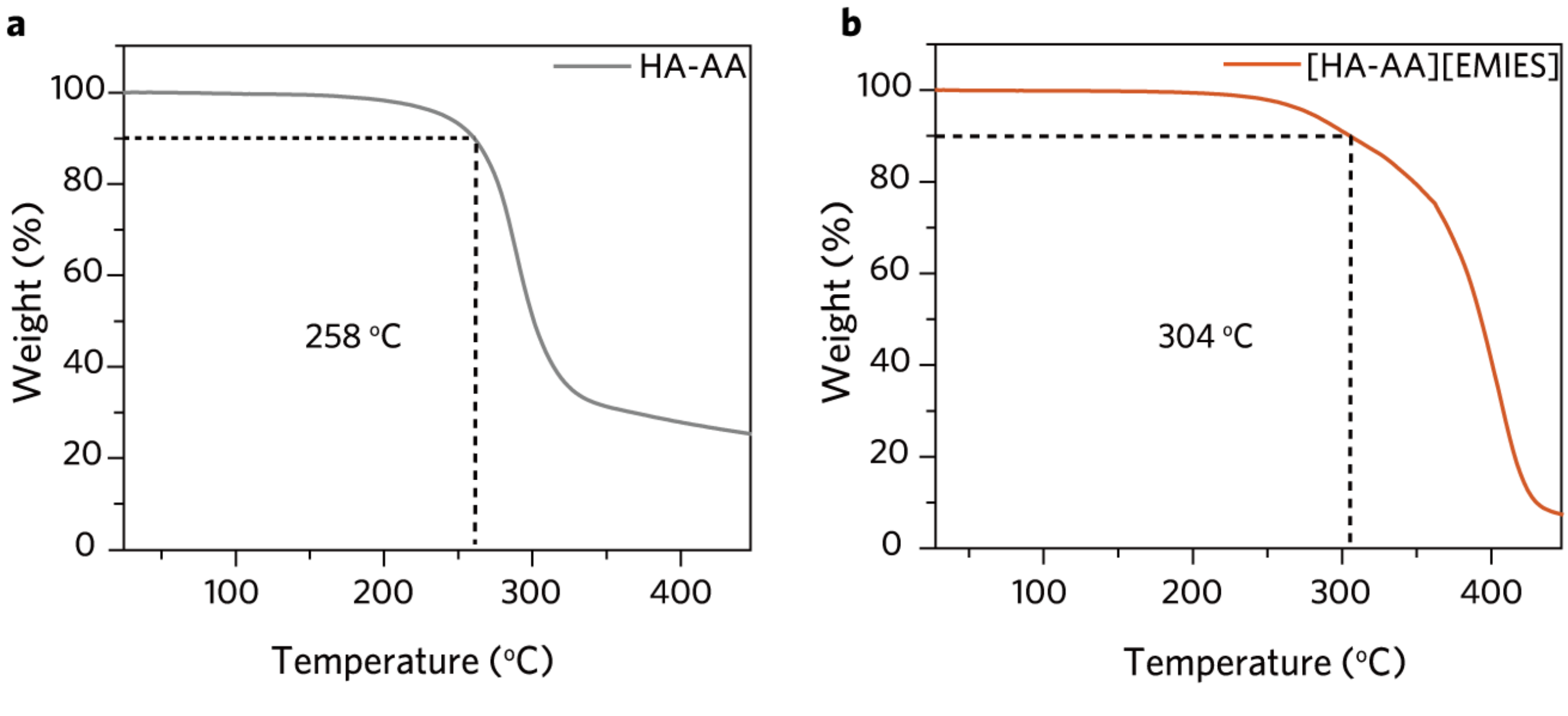

Figure S2. TGA curves of HA-AA (**a**) and [HA-AA][EMIES] (**b**).

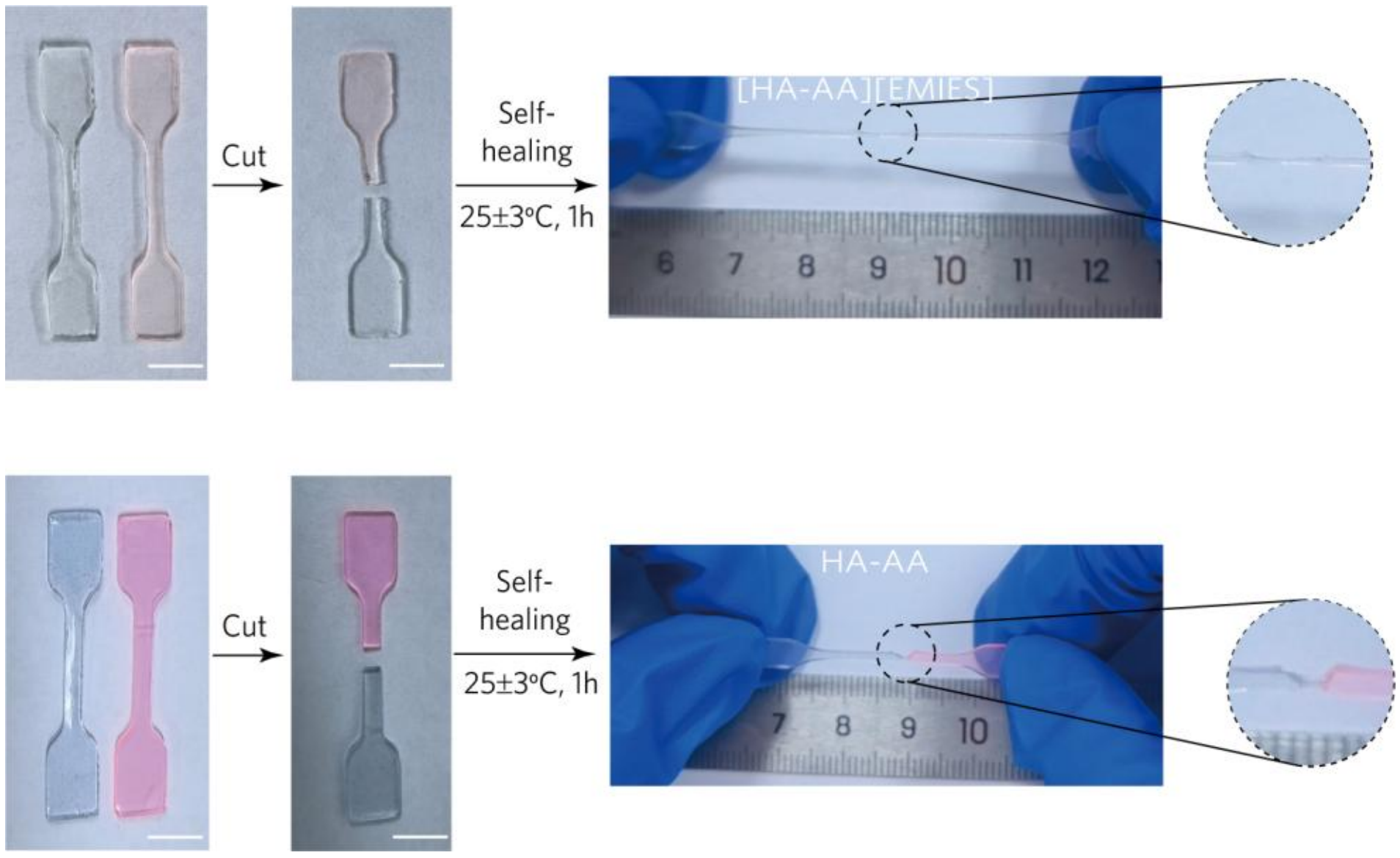

Figure S3. Demonstration of self-healing behaviors of HA-AA and [HA-AA][EMIES] at room temperature. The circular insets show the magnified views of the healed interfaces after stretching. The scale bar in the dog-bone-shaped samples (dyed blue and red) is 1 cm.

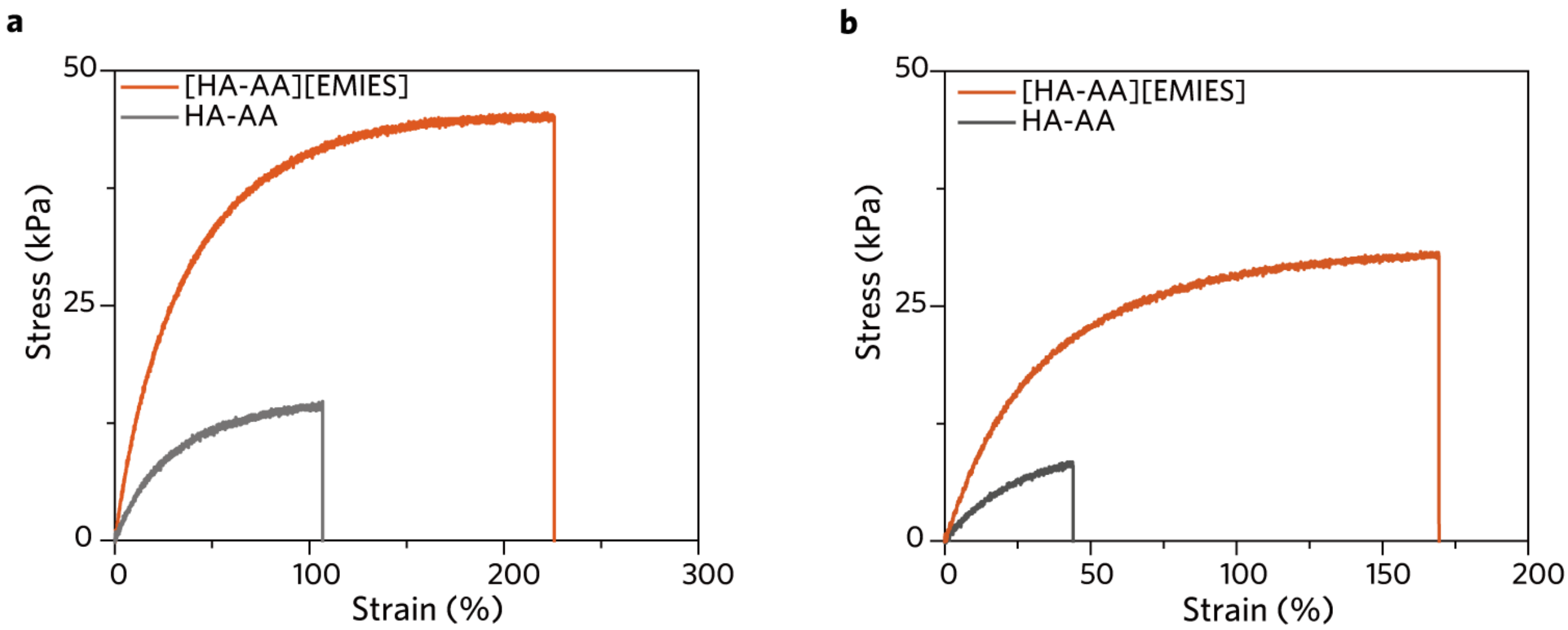

Figure S4. Tensile-strain curves of healed [HA-AA][EMIES] and HA-AA after 1 h self-healing at 60ºC (**a**) and room temperature (**b**)**.** The stretched deformation rate is 5 mm min^{-1}.

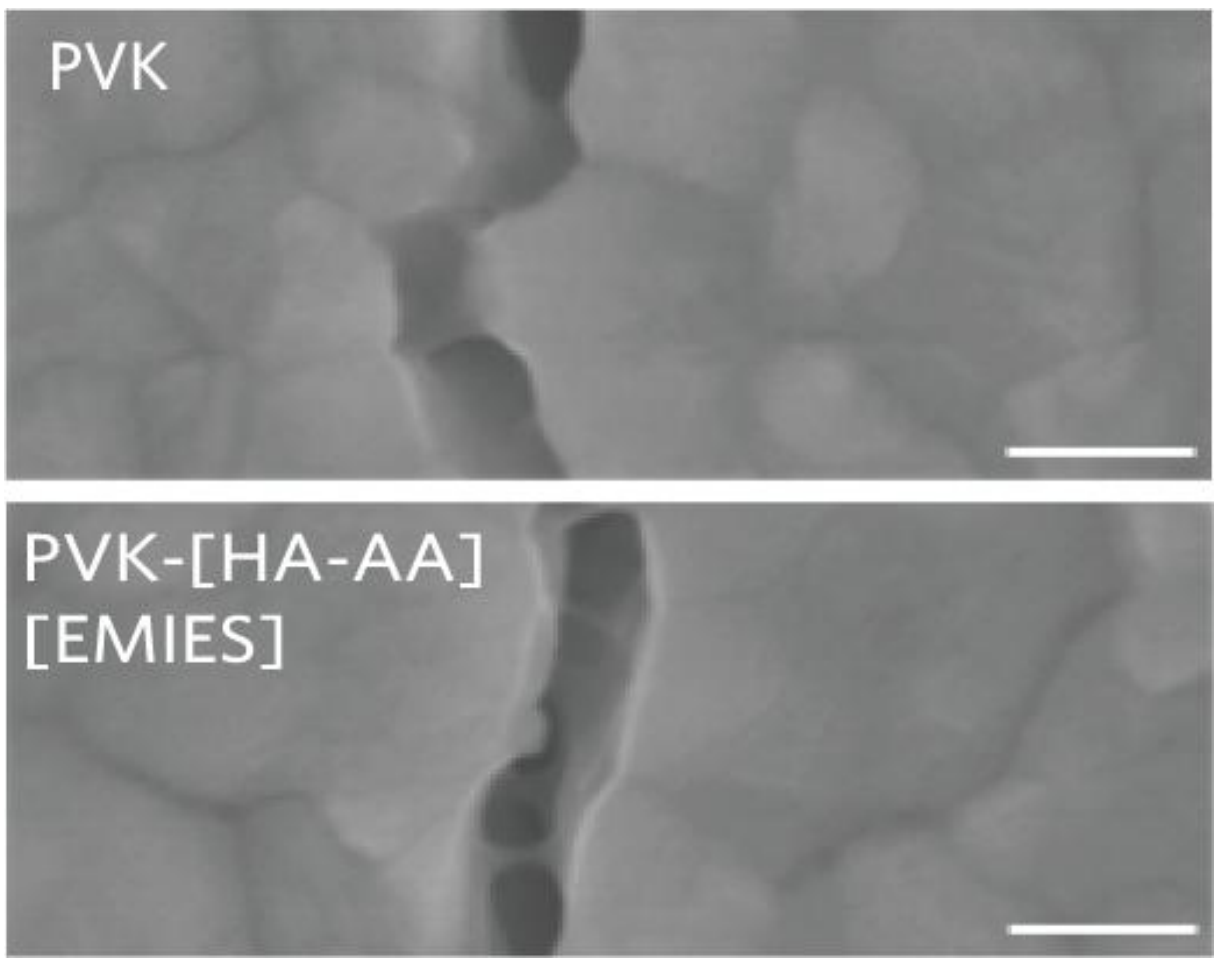

Figure S5. High-resolution SEM images of PVK and PVK-[HA-AA][EMIES]. The bottom image shows [HA-AA][EMIES] at the GBs of the perovskite film. Scale bar: 1 μm.

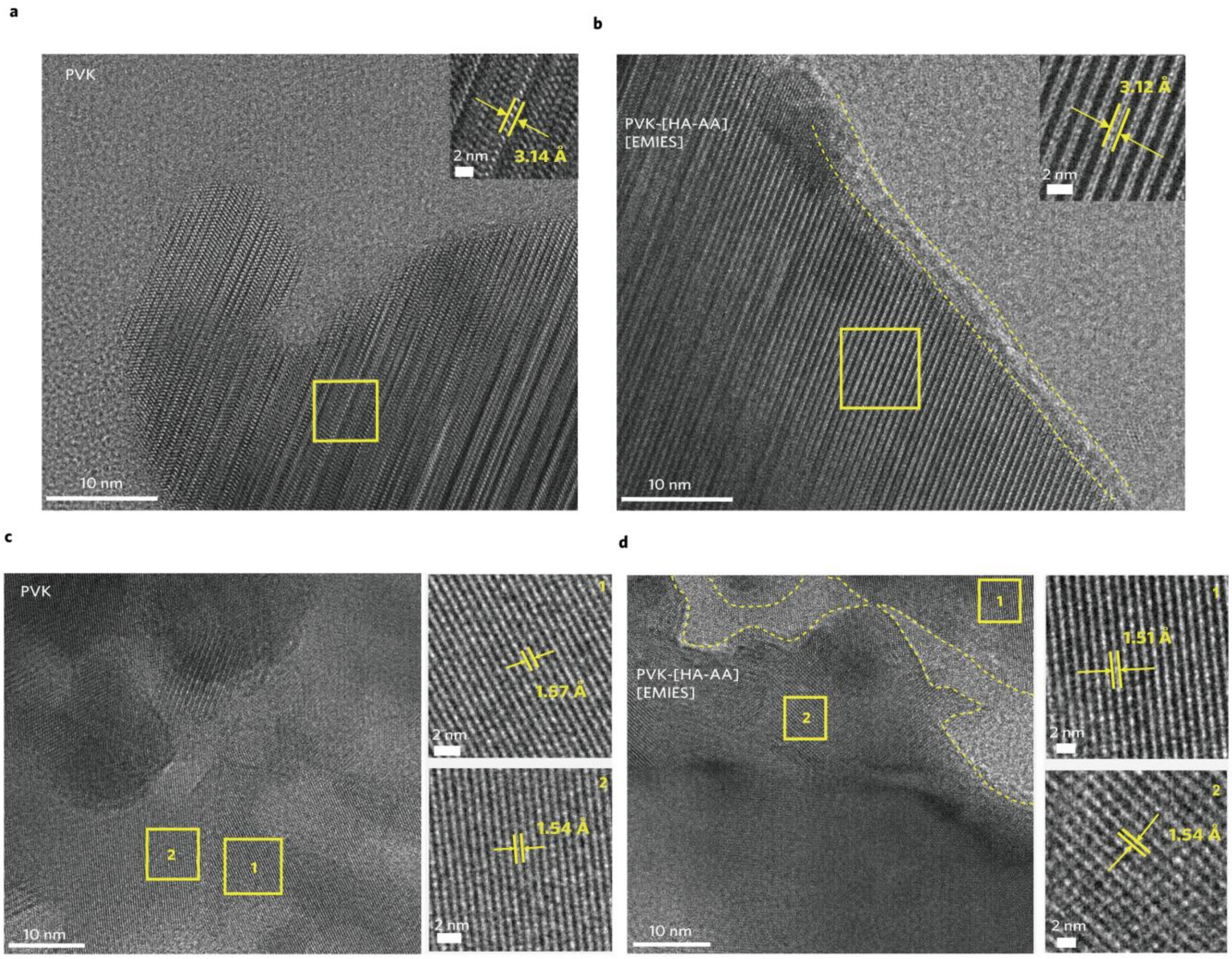

Figure S6. HR-TEM characterizations of PVK and PVK-[HA-AA][EMIES]. (**a**) Edge region of PVK without an obvious amorphous phase. (**b**) Edge region of PVK-[HA-AA][EMIES], showing a

continuous amorphous interfacial phase highlighted by the yellow dashed lines. (**c**) Internal grain boundary of PVK with clear crystalline lattice fringes. (**d**) Internal grain boundary of PVK-[HA-AA][EMIES], showing an amorphous phase between adjacent crystalline grains. The insets show the enlarged views of yellow-boxed region. Lattice spacings of 1.51-1.57 Å and 3.12-3.14 Å are assigned to the (200) and (100) reflections of cubic α-$FAPbI_3$, respectively.

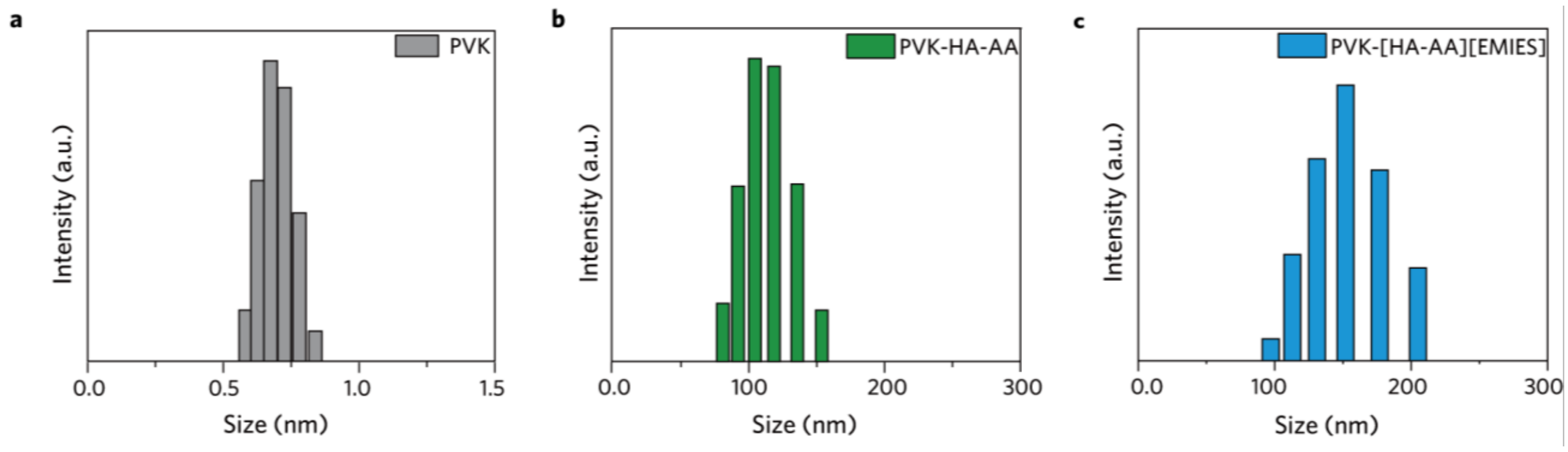

Figure S7. DLS measurements of PVK (**a**), PVK-HA-AA (**b**) and PVK-[HA-AA][EMIES] (**c**).

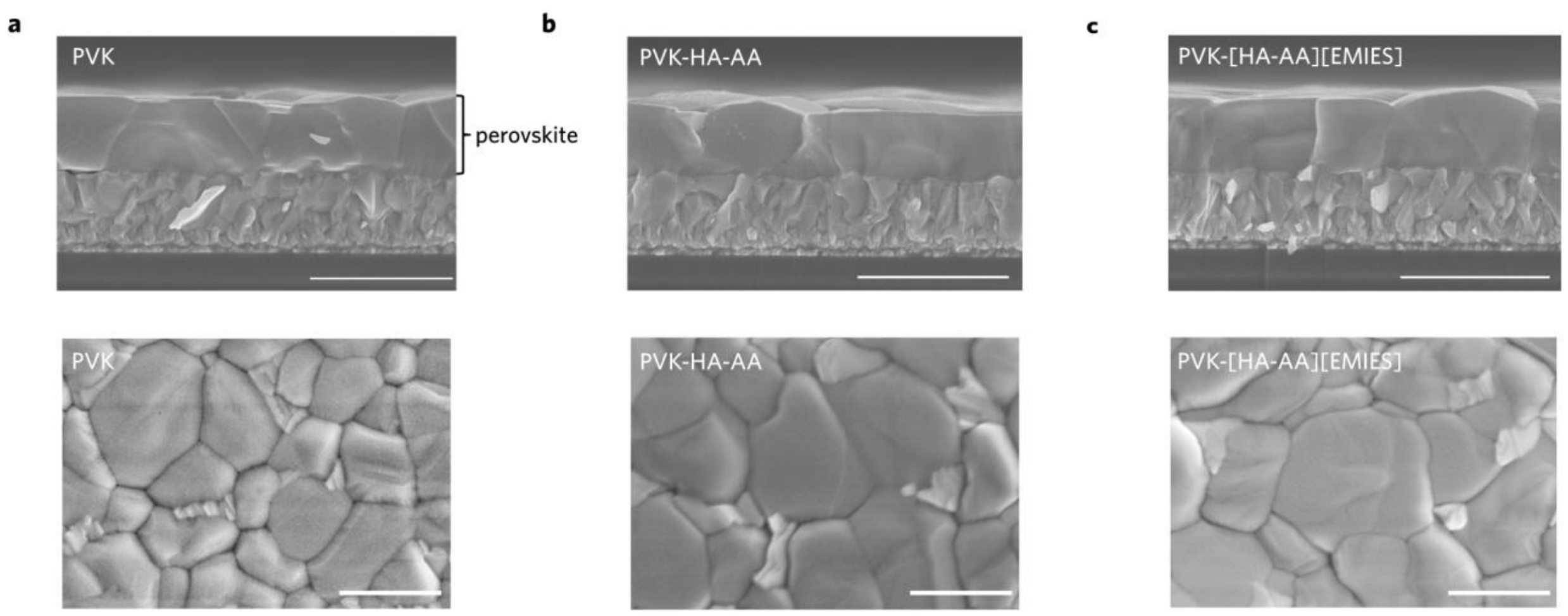

Figure S8. Cross-section SEM (above) and scanning SEM (bottom) images of perovskite films. The scale bar are 1 μm and 500 nm in the above and below images, respectively.

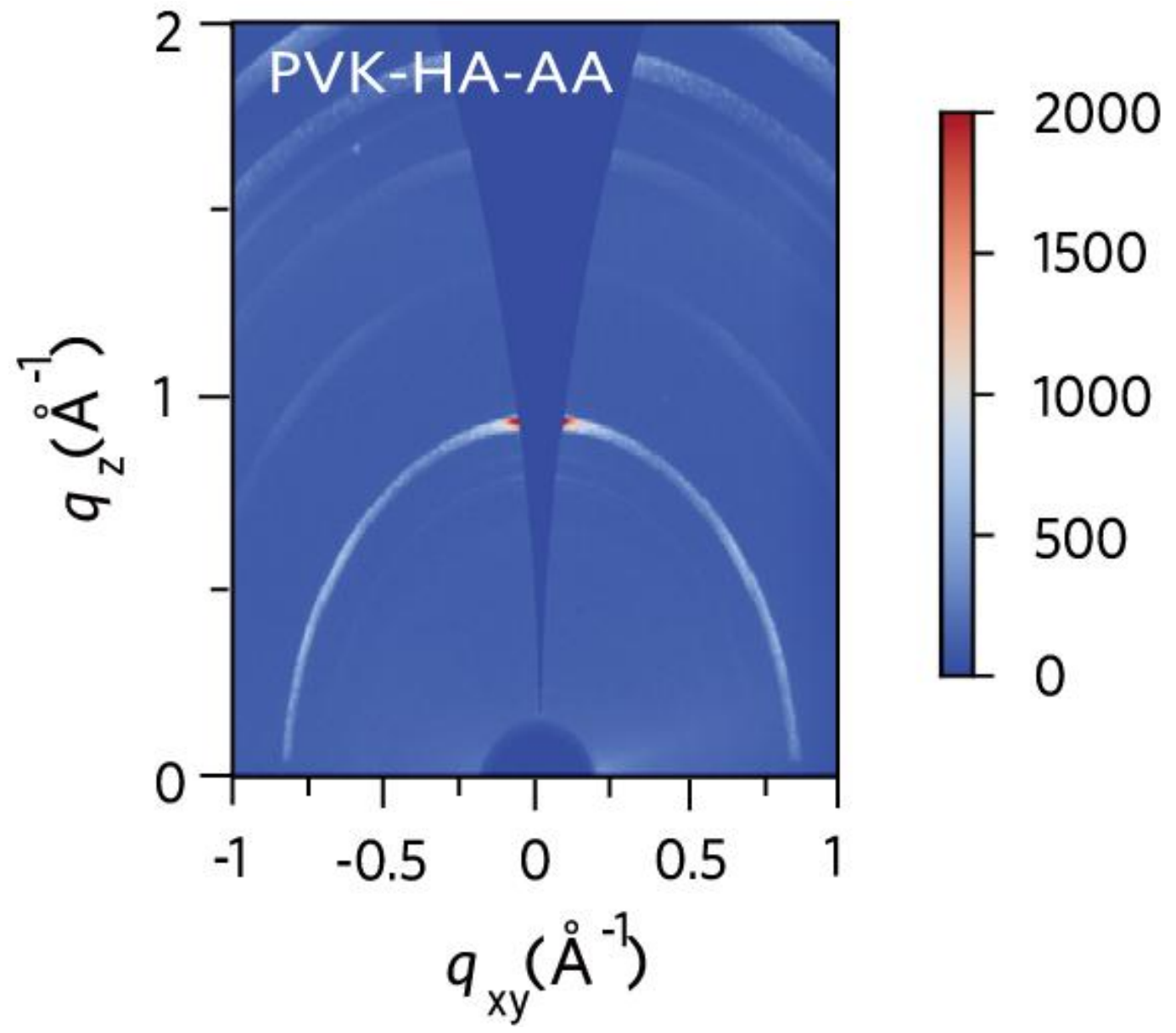

Figure S9. GIWAXS profile of PVK-HA-AA.

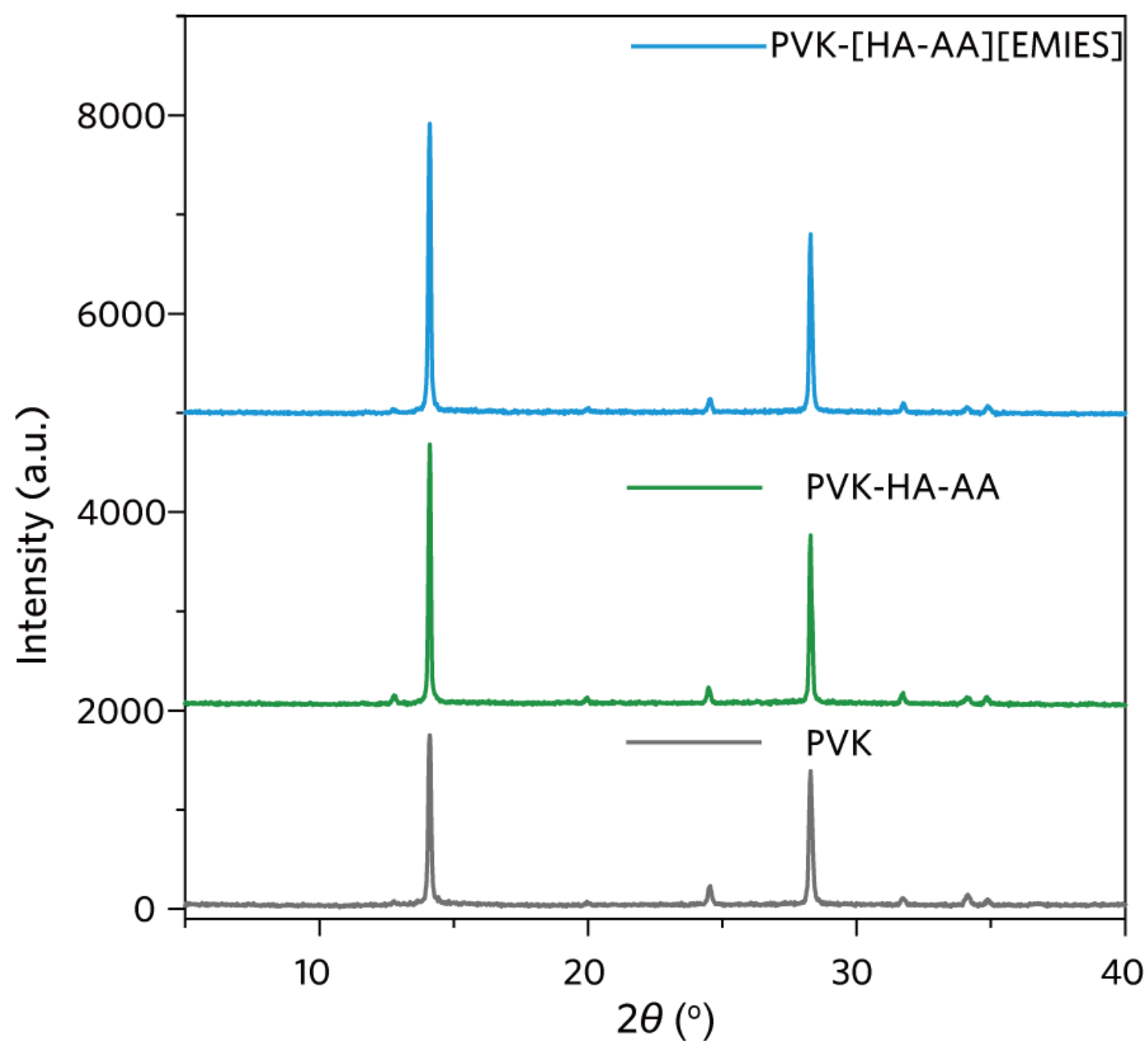

Figure S10. XRD patterns of PVK, PVK-HA-AA and PVK-[HA-AA][EMIES].

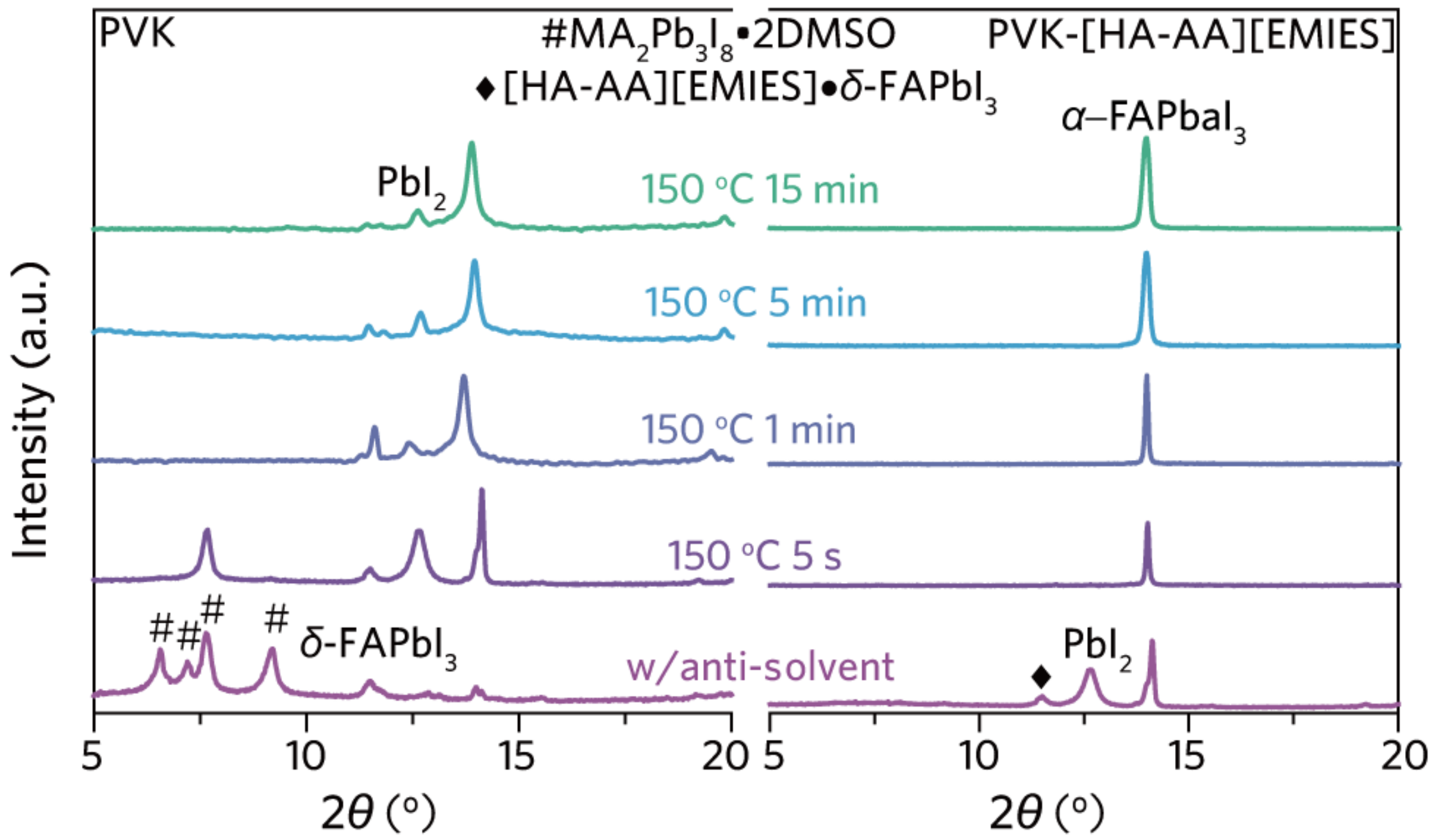

Figure S11. In-situ tracking of XRD of perovskite films (PVK and PVK-[HA-AA][EMIES]) during two processes: wet perovskite films with (*w*) anti-solvent during spin-coating, and then annealed at 150°C for various time.

Table S1. Summary of *S* and *A* parameters for nano-indentation measurements.

Sample	*S* (μN nm^{-1}) [a]	*A* (nm^2)[b]
PVK	18.67	190048
PVK-HA-AA	14.82	231306
PVK-[HA-AA][EMIES]	15.92	378151

[a]*S* represents the contact stiffness obtained from the initial slope of the unloading curve. [b]*A* represents the projected contact area between indenter and perovskite film.

Table S2. The related parameters of every layer for the finite element simulation.

Layer	Thickness (μm)	Young's modulus (Gpa)
ITO	0.08	210
SnO_2	0.03	82.4
PVK	0.55	37.95
PVK-HA-AA	0.55	27.30
PVK-[HA-AA][EMIES]	0.55	22.94

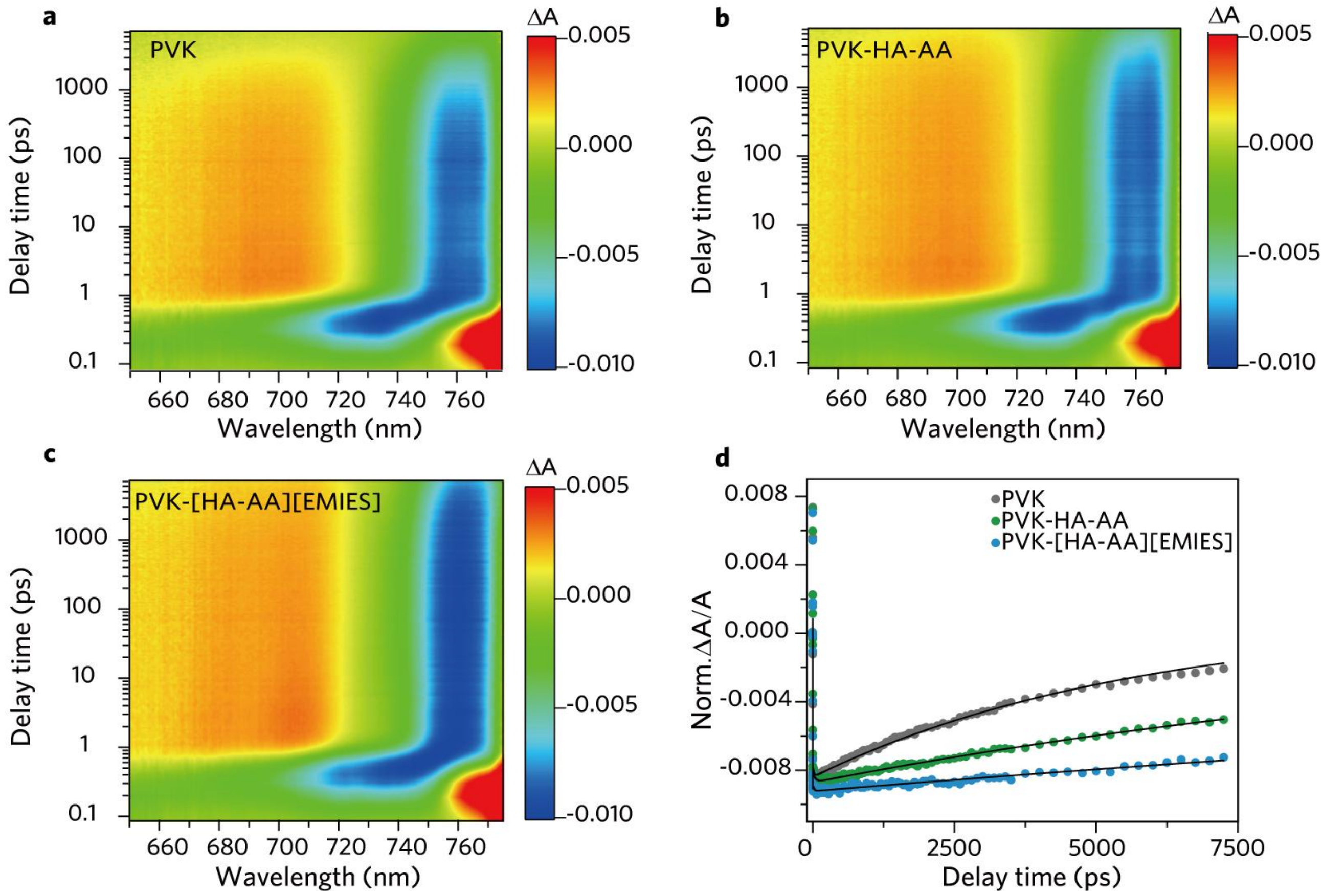

Figure S12. (**a-c**) Pseudocolor representation of the TA spectra of PVK (**a**), PVK-HA-AA (**b**) and PVK-[HA-AA][EMIES] (**c**). **d** Normalized bleaching kinetics at 767 nm for perovskite films after excitation at 400 nm. The pump fluence for all samples is 0.48 μJ cm^{-2}.

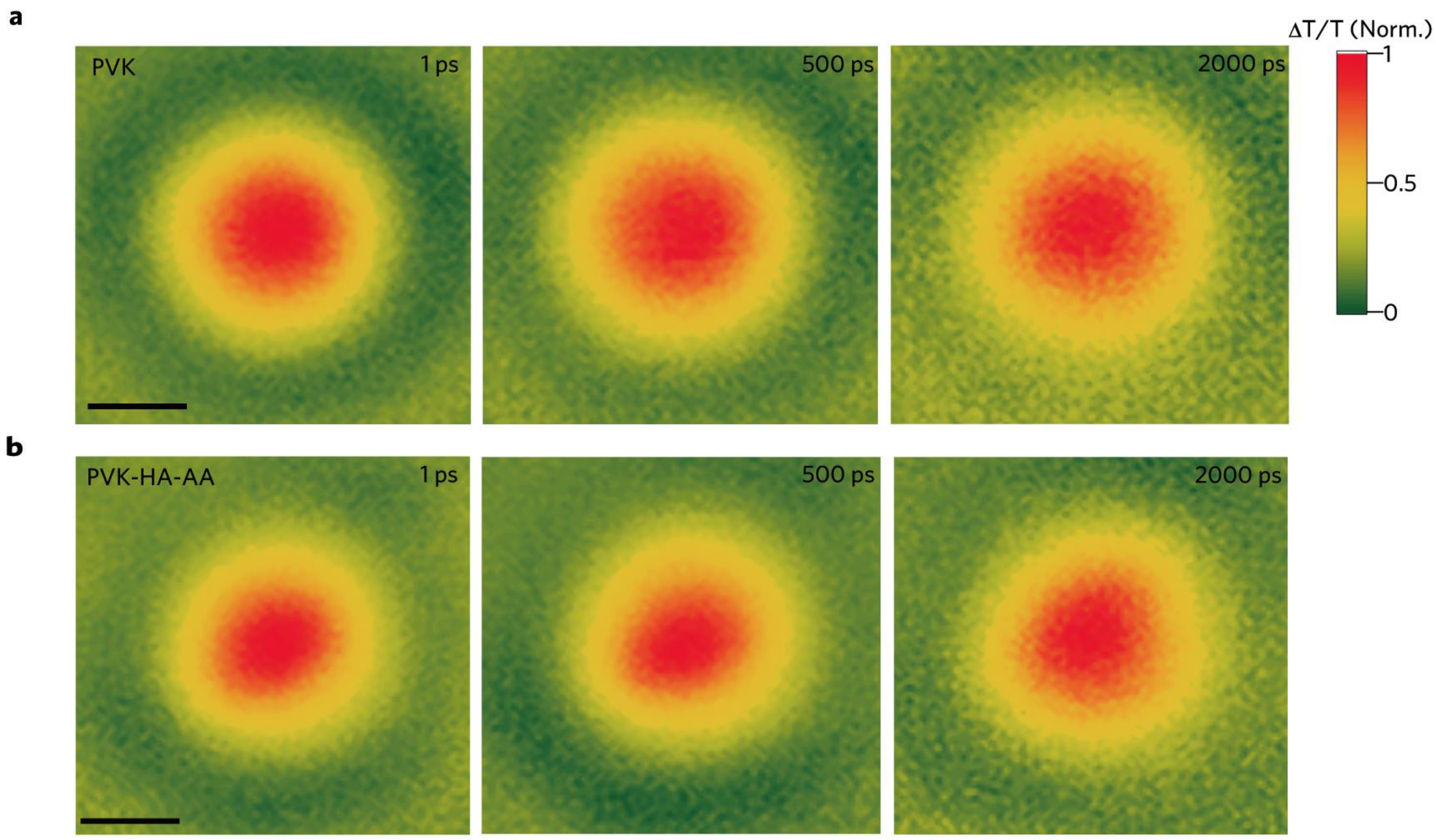

Figure S13. Two-dimensional TAM images of PVK (**a**) and PVK-HA-AA (**b**) at different delay time under a pump fluence of 1.05 μJ cm^{-2}. The color scale represents the intensity of pump-induced ΔT of the probe, and every image has been normalized by peak value. The scale bar is 1 μm. The probe wavelength is 767 nm.

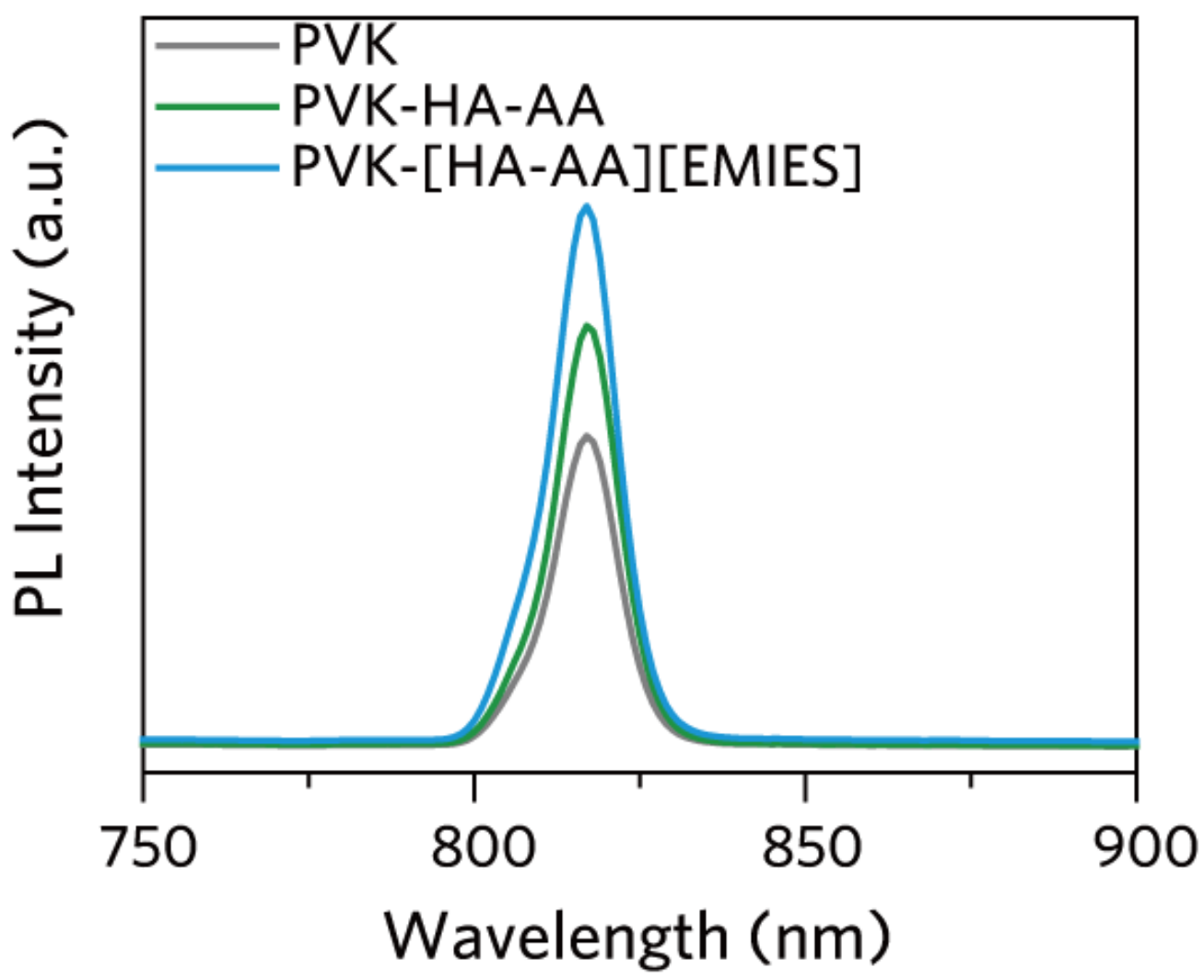

Figure S14. PL spectra of PVK, PVK-HA-AA and PVK-[HA-AA][EMIES].

Table S3. Fitting parameters obtained from the simulation of TAM data of perovskite films.

Sample	D (cm^2 s^{-1})	τ_{avg} (ns)	L (μm)
PVK	0.19	228.19	4.13
PVK-HA-AA	0.27	487.21	5.28
PVK-[HA-AA][EMIES]	0.28	1109.79	8.07

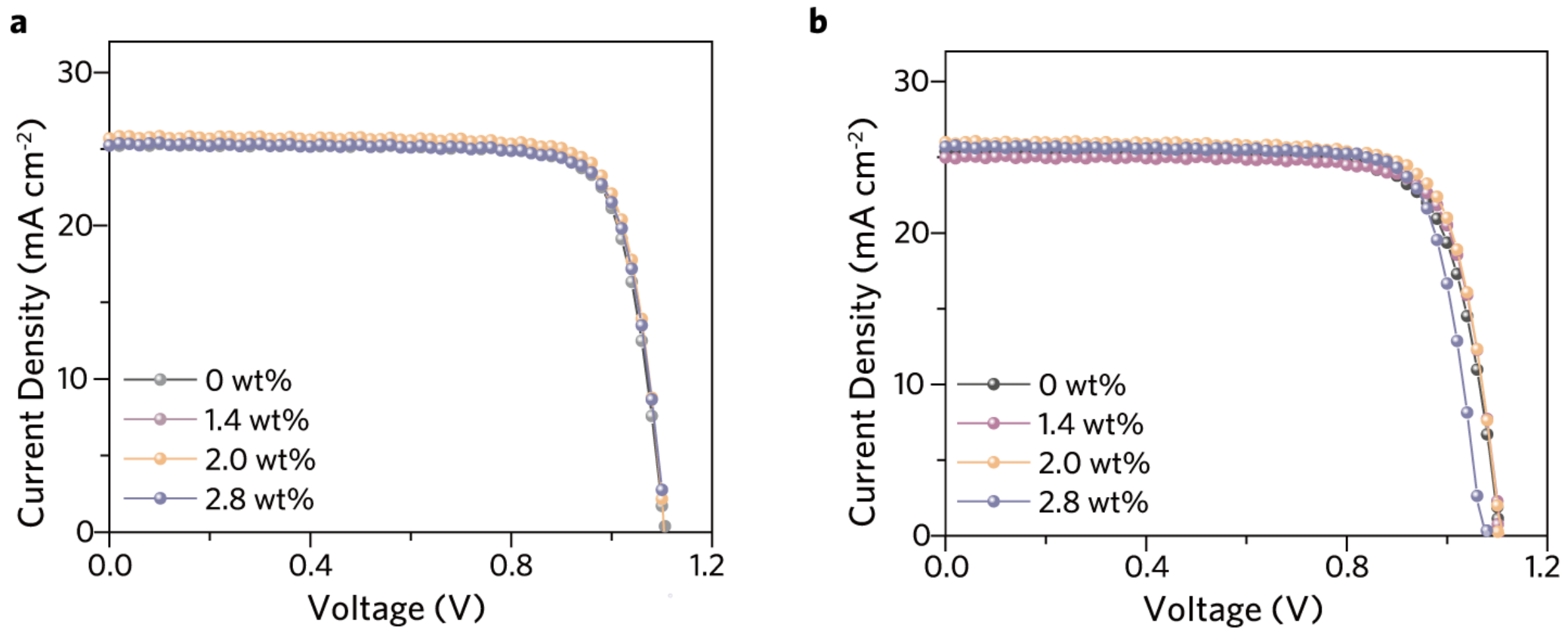

Figure S15. *J-V* curves of the devices with different HA-AA (**a**) and [HA-AA][EMIES] concentration (**b**). HA-AA and [HA-AA][EMIES] were completely dissolved into the perovskite precursor solution respectively. The addition amounts of both were 0 wt%, 1.4 wt%, 2.0 wt% and 2.8 wt% of the PbI_2 component.

Table S4. The best photovoltaic parameters of devices under standard AM 1.5G illumination (100 mW cm^{-2}).

Sample	V_{OC} (V)	J_{SC} (mA cm^{-2})	FF (%)	PCE (%)
PVK	1.16	25.54	83.06	24.83
PVK-HA-AA	1.18	25.97	83.08	25.57
PVK-[HA-AA][EMIES]	1.19	26.12	85.28	26.45

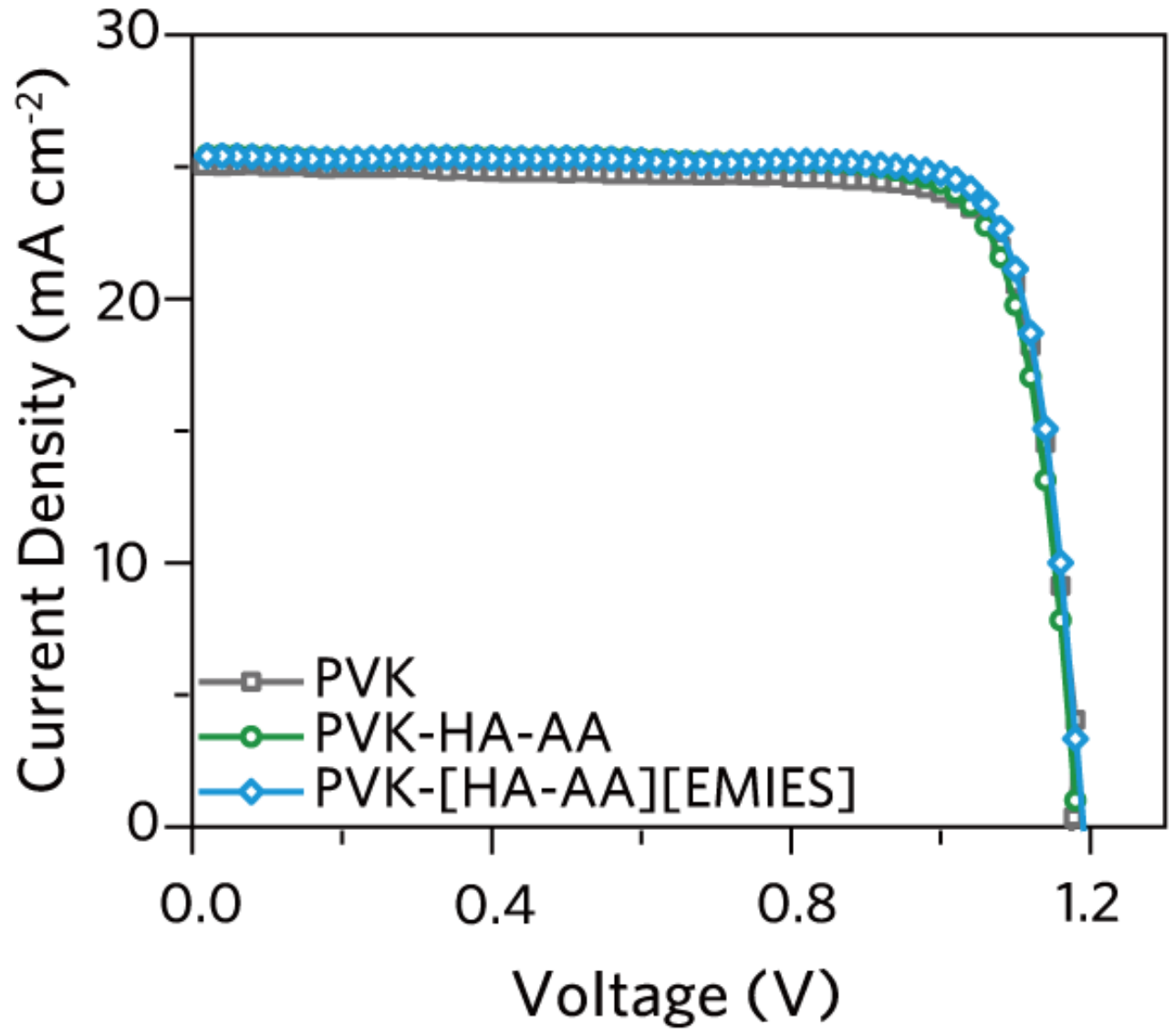

Figure S16. Optimal *J-V* curves for the *f*-PSCs based on PVK, PVK-HA-AA and PVK-[HA-AA][EMIES].

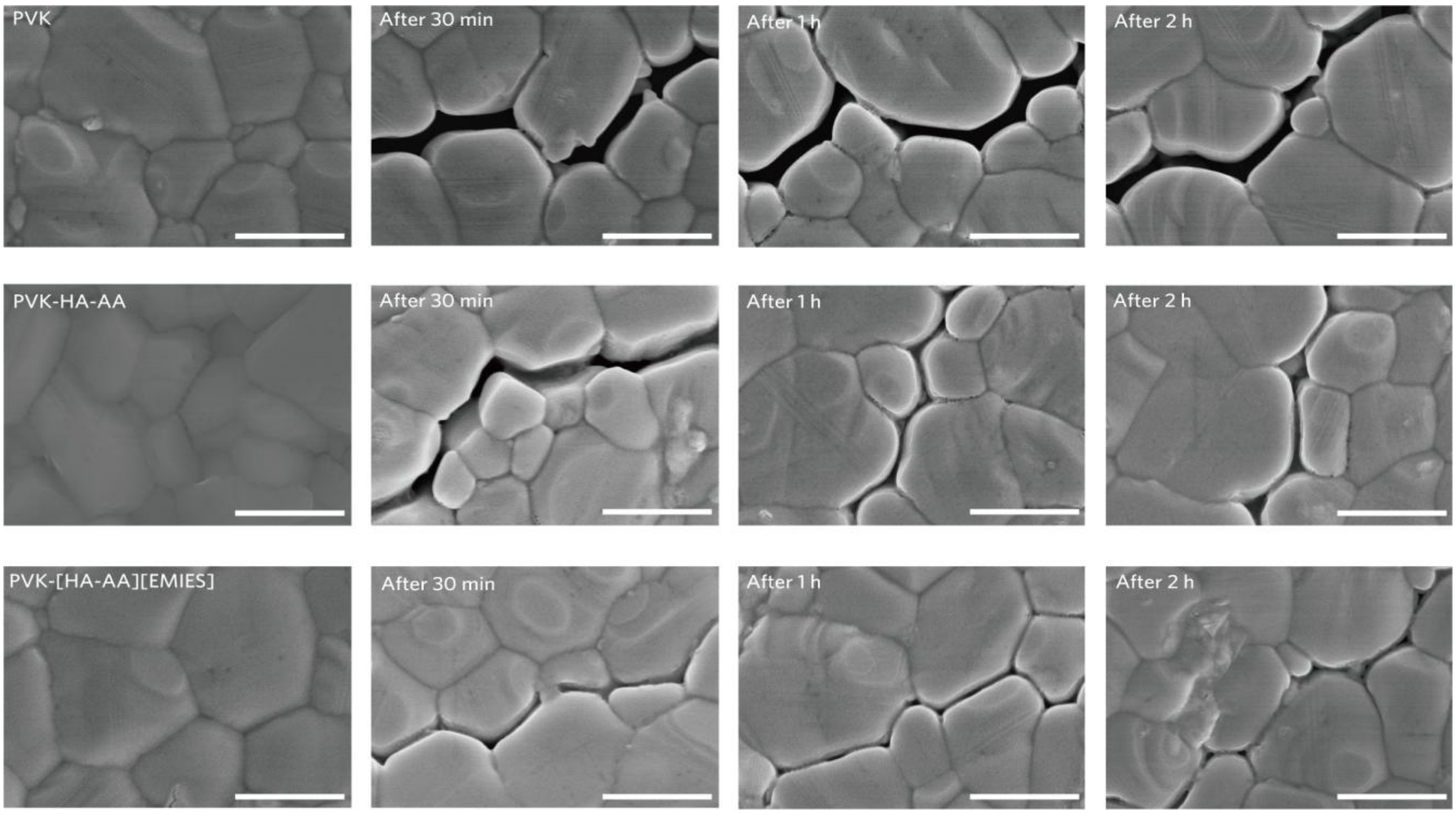

Figure S17. SEM images showing the time-dependent recovery of bending-induced cracks in PVK, PVK-HA-AA, and PVK-[HA-AA][EMIES] films. The leftmost column shows the initial surface morphologies before bending. After 10,000 bending cycles, the film morphologies were obtained with recovering for 30 min, 1 h, and 2 h, respectively. The scale bar is 1 μm.

Table S5. The best photovoltaic parameters of *f*-PSCs under standard AM 1.5G illumination (100 mW cm^{-2}).

Sample	V_{OC} (V)	J_{SC} (mA/cm^2)	FF (%)	PCE (%)
PVK	1.18	24.79	79.60	23.40
PVK-HA-AA	1.18	25.43	81.46	24.51
PVK-[HA-AA][EMIES]	1.19	25.41	83.18	25.14

Table S6. The defect densities of electron-only devices with a configuration of FTO/SnO_2 /perovskite/PCBM/Ag.

Sample	V_{TFL} (V)[a]	N_{trap}(cm^{-3})[b]
PVK	1.0	4.42×10^{15}
PVK-HA-AA	0.6	2.65×10^{15}
PVK-[HA-AA][EMIES]	0.3	1.32×10^{15}

[a] V_{TFL}: trap-filled-limit voltage. [b] N_{trap}: trap-state density.
